# Repurposing Drugs for Senotherapeutic Effect: Potential Senomorphic Effects of Female Synthetic Hormones

**DOI:** 10.3390/cells13060517

**Published:** 2024-03-15

**Authors:** Laura R. Bramwell, Ryan Frankum, Lorna W. Harries

**Affiliations:** RNA-Mediated Mechanisms of Disease Group, Department of Clinical and Biomedical Sciences (Medical School), Faculty of Health and Life Sciences, University of Exeter, Exeter EX2 5DW, UK; l.bramwell2@exeter.ac.uk (L.R.B.); rf402@exeter.ac.uk (R.F.)

**Keywords:** senescence, structure–function screen, synthetic hormone, sex differences, sex-specific, senomorphic

## Abstract

Repurposing previously approved drugs may fast track the route to the clinic for potential senotherapeutics and improves the inefficiency of the clinical drug development pipeline. We performed a repurposing screen of 240 clinically approved molecules in human primary dermal fibroblasts for their effects on *CDKN2A* expression. Molecules demonstrating effects on *CDKN2A* expression underwent secondary screening for senescence-associated beta galactosidase (SAB) activity, based on effect size, direction, and/or molecule identity. Selected molecules then underwent a more detailed assessment of senescence phenotypes including proliferation, apoptosis, DNA damage, senescence-associated secretory phenotype (SASP) expression, and regulators of alternative splicing. A selection of the molecules demonstrating effects on senescence were then used in a new bioinformatic structure–function screen to identify common structural motifs. In total, 90 molecules displayed altered *CDKN2A* expression at one or other dose, of which 15 also displayed effects on SAB positivity in primary human dermal fibroblasts. Of these, 3 were associated with increased SAB activity, and 11 with reduced activity. The female synthetic sex hormones—diethylstilboestrol, ethynyl estradiol and levonorgestrel—were all associated with a reduction in aspects of the senescence phenotype in male cells, with no effects visible in female cells. Finally, we identified that the 30 compounds that decreased *CDKN2A* activity the most had a common substructure linked to this function. Our results suggest that several drugs licensed for other indications may warrant exploration as future senotherapies, but that different donors and potentially different sexes may respond differently to senotherapeutic compounds. This underlines the importance of considering donor-related characteristics when designing drug screening platforms.

## 1. Introduction

Senescence is a hallmark of ageing, and an emerging therapeutic target [1,2]. Senescence may appear as part of natural development, but during ageing, it is induced by replicative exhaustion or by cellular stressors such as DNA damage, oncogenes, and other forms of cellular stress [3,4,5,6,7]. Despite the original definition that senescence is irreversible, recent research indicates that the senescence phenotypes can be reversed by some classes of drugs [8,9]. Senotherapeutics (compounds that target senescence) include those that attenuate the deleterious characteristics of senescent cells (senomorphics) and drugs that cause preferential lysis of senescent cells (senolytics) [8,9,10]. Clearance of senescent cells significantly extends lifespan, improves mobility and fur condition in mouse models of progeria, and improves multiple aspects of functionality in aged wild-type mice [11,12,13,14,15]. Senolysis has also been seen to confer additional health benefits in humans; combinations of the senolytic drugs dasatinib and quercetin compounds are currently in trials for diabetic kidney disease and idiopathic pulmonary fibrosis (IPF) [16,17]. However, some initial results from senolytic trials also show potential adverse effects, e.g., hypoglycaemia [18].

Attenuating the senescent state using senomorphic approaches may also be useful. It is possible to uncouple features of senescence, such as reversal of senescence-associated beta galactosidase (SAB) staining from other aspects such as proliferation; such effects are often dose-dependent [19]. The ideal senotherapeutic candidate would be able to reverse senescence and attenuate the senescence-associated secretory phenotype (SASP) (a senomorphic effect), but would not necessarily elicit re-entry to cell cycle, since rejuvenated cells may still carry a mutation load. Conversely, any compounds that are identified as increasing senescence might represent potential oncodrugs. Forcing cancerous cells to enter a senescent state might provide a better tolerated oncotherapeutic approach and provide an opportunity to selectively target the resulting cells with senolytic drugs.

It is likely that some known and licensed drugs have some senomorphic or senolytic capacity. The drug development pipeline is inefficient, with only 15.3% of drugs in phase 1 clinical trials in the US advancing to gain FDA-approval [20]. Repurposing drugs which are already approved for clinical use represents a tactic which avoids the problems with the leaky pipeline of drug development. For example, trametinib, a MEK inhibitor currently used as a cancer treatment, exhibits a biphasic dose response, affecting different aspects of senescence depending on dose [21]. Panels of small molecules for drug repurposing studies can be procured and customized commercially, giving plenty of opportunities to adapt drug repurposing screens for different indications. 

Bioinformatic approaches can also be used to complement wet laboratory screening. Structure–function associations may be of particular interest in the context of a screen for senescence. If a certain structure is associated with a senomorphic or senolytic function, then this provides an opportunity to identify potentially useful compounds from public drug databases by screening them for the structure. This strategy could offer the discovery of novel drugs in a quicker way than traditional pharmaceutical discovery processes. Similarly, any structural association with specific senescence related functions may provide mechanistic insight into the cellular processes at hand. 

We aimed to screen a range of compounds for effects on aspects of the senescence phenotype using in vitro screens in primary human dermal fibroblasts and bioinformatic structure–function analysis. We identified several existing clinically approved molecules as having capacity to attenuate aspects of the senescence phenotype in a sex-specific manner. Finally, we have worked up a structure–function screening pipeline and identified a molecular substructure that is associated with alterations in *CDKN2A* expression (a biomarker of senescence) or SAB positivity. Our work indicates that repurposing studies augmented by bioinformatic or machine learning approaches may prove a rich vein of research for the identification of new classes of senotherapeutic molecules, but donor characteristics, such as sex and individual genetics, can influence senescence outcome and should be accounted for in study design.

## 2. Materials & Methods

### 2.1. Drug Panel, Screen Design and Preparation

**Figure 1:**
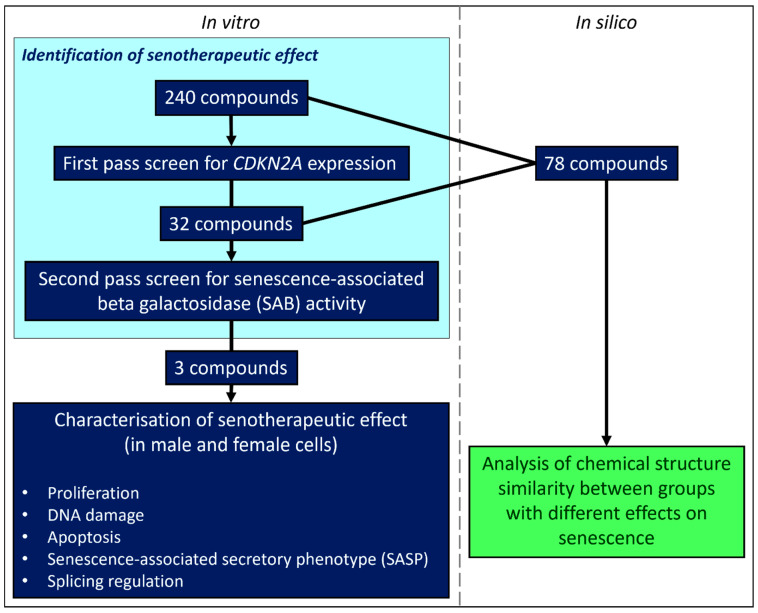
Flowchart describing experimental design.

A selection of 240 compounds were chosen from the MedChemExpress FDA-Approved Drug Library Plus panel of 2278 compounds (MedChemTronica, Stockholm, Sweden). We selected drugs that target known senescence/cell fate pathways, that cover a variety of other cellular functions (including apoptosis and autophagy), and a variety of commonly prescribed or household medicines. Drug identities and targets are given in Supplementary Table S1 and Supplementary Figure S1 (summary graphs were produced using Microsoft Office, Redmond, WA, USA). Most compounds are FDA-approved with the remainder approved by the EMA or other countries. Compounds were all supplied at 10 mM concentrations in DMSO by the manufacturer. Concordant with similar screens [19,22], compounds were diluted to 1 µM or 10 µM in Gibco™ ultrapure RNase/DNase free water suitable for preparation of cell culture media and laboratory reagents (A1287301, Gibco™, Billings, MT, USA). Vehicle DMSO controls were prepared in the same manner. On the basis of our first pass results, an additional synthetic female hormone (Levonorgestrel; also known as D-Norgestrel, MedChemTronica, Stockholm, Sweden) which targets the progesterone receptor was also added to secondary screen. A flow chart (produced using Microsoft Office and Biorender.com, Toronto, Canada) illustrating our screening approach is given in Figure 1 and the Graphical Abstract.

### 2.2. Cells Used in This Study

Normal human dermal fibroblast (nHDF) cells from one male and one female donor were commercially sourced with full ethical permission granted at source (Promocell, Heidelberg, catalogue number C-12302, lot numbers 445Z026.3 (male) and 467Z026.3 (female)). Both donors were Caucasian. The male donor was 36 years old at the time of donation, and the female donor was 28 years old. The cells were taken from the abdomen of the male donor, whereas the female donor’s cells were taken from the breast. Cells were grown in DMEM 1 g/L glucose + phenol red (31885023, Gibco™, Billings, MT, USA), 10% human serum (H3667, Sigma-Aldrich, St. Louis, MO, USA) and 1% 10,000 U/mL penicillin—10,000 µg/mL streptomycin (15140122, Gibco™, Billings, MT, USA). Cells were grown in antibiotic–free media for 48 to 72 h before seeding, and all treatments were performed without the presence of antibiotics in the medium.

### 2.3. Primary Screen

#### 2.3.1. Tissue Culture and Drug Treatment Conditions

Male nHDF cells had average cumulated population doublings (cPDL) of 38.91 (range of 34.41–40.13 cPDL) at the time of seeding for the primary screen. For this, cells were seeded out in 96-well plates at 6000 cells per well (a density of 1880 cells/cm^2^) and treated 72 h after seeding. Dose and incubation times were informed by previous work from our research group and the literature discussed in the introduction [19,22,23]. On the day of treatment, the medium was removed and replaced with 135 µL of fresh medium and 15 µL of the appropriate stock solution of each drug or control. Doxorubicin was included as a known inducer of senescence. The drug or control was applied for 24 h before two washes in DPBS (catalogue number 14190136, Gibco™, Billings, MT, USA) and performing the RNA extraction.

#### 2.3.2. Quantification of *CDKN2A* Expression

RNA was extracted from treated cells using the PureLink™ Pro 96 RNA Purification Kit (catalogue number 12173–011A, Fisher Scientific, Pittsburgh, PA, USA) according to manufacturer’s instructions and eluted in a volume of 45 µL of RNase-free water. RNA quality and quantity was sampled using the Thermo Scientific™ Nanodrop 8000 Spectrophotometer (ThermoFisher, Waltham, MA, USA). Two test plates had compromised RNA quality and were excluded from the analysis. The maximum RNA volume possible based on reaction volume constraints was reverse-transcribed using the High-Capacity cDNA Reverse Transcription Kit (catalogue number 4368813, ThermoFisher, Waltham, MA, USA) according to the manufacturer’s instructions. Reverse transcription was performed on the Applied Biosystems™ Veriti™ 96-Well Fast Thermal Cycler platform. Cycling conditions were: 25 °C for 10 min, 37 °C for 120 min, 85 °C for 5 min and a 4 °C hold step. Real-time quantitative PCR (RT-qPCR) reactions were carried out on the Quantstudio 12K platform (Applied Biosystems™, Birchwood, UK) as 5 µL reactions on 384-well plates. Cycling conditions were: 50 °C for 2 min, 95 °C for 10 min, followed by 50 cycles of 95 °C for 15 s and 60 °C for 1 min. Each reaction contained 1 µL of cDNA product, 900 nM each primer and 250 nM probe and TaqMan™ Universal Mastermix II. *CDKN2A* expression was assessed using the Hs00923894_m1 TaqMan™ Gene Expression Assay (FAM) (catalogue 4331182, ThermoFisher, Waltham, MA, USA). Endogenous housekeeper control genes were *PGK1* (assay ID HS99999906_m1), *PPIA* (assay ID Hs04194521_s1) and *UBC* (assay ID Hs05002522_g1), empirically determined to represent the most stable baseline accordingly to the RefFinder webtool [24]. All were procured from ThermoFisher (Waltham, MA, USA). Assays were performed in two biological and three technical replicates for each compound/control at both treatment doses. Relative gene expression levels were calculated using the comparative C_T_ technique relative to the geometric mean expression level of the three housekeeping genes [25]. Levels were normalized to the average of the vehicle control on each plate and were expressed as natural log to aid against skew of data. The mean ± three standard deviations was used to provide upper and lower bounds for prioritization of compounds for follow up. 

### 2.4. Secondary Screen

To assess the induction of senescence, experiments were carried out using early passage male cells (cPDL = 32.69), whereas work to assess potential reduction in senescent cell load was carried out using later passage cells (cPDL = 40.77–43.2). Later passage cells were assessed at the point that they had slowed to half their original division speed. For assessment of SAB activity, cells were seeded in 12-well plates at an average seeding density of 6226 cells/cm^2^. Cells were grown for 24 to 48 h before treatment as in the primary screen and each compound was applied for 24 h prior to assessment of SAB activity, which was carried out using the Senescence Cells Histochemical Staining Kit (Merck, Gillingham, UK), according to manufacturer’s instructions. Five images per biological replicate were imaged at 10 × magnification using a Zeiss AxioCam ERC55 PrimoVert microscope and later counted manually using ImageJ 1.47v software (US National Institute of Health, Bethesda, MD, USA) [26]. Differences in SAB staining between test compounds and controls were assessed by one-way ANOVA with an uncorrected Fisher’s LSD *post hoc* test and graphed using GraphPad Prism version 9.4.1 for Windows (GraphPad Software, San Diego, CA, USA, www.graphpad.com, accessed on 24 January 2024).

### 2.5. In Depth Characterization of Female Synthetic Hormone Compounds

#### 2.5.1. Tissue Culture and Dosing Regime

Based on the results of our primary and secondary screen, we selected three female synthetic hormones for follow up due to evidence of effects on senescence. Cells for this work had an average cPDL of 39.46 at the time of seeding for male cells and 33.68 for female cells, and were assessed as being late passage at the point that they had slowed to half their original division speed. Cells were seeded at approximately 7200 cells/cm^2^ in a 12-well plate for the SAB assay, at ~6000 cells/cm^2^ in a 12-well plate on 13 mm coverslips for immunocytochemical staining, at ~7000 cells/cm^2^ in a 24-well plate on 13 mm coverslips for the TUNEL assay experiments and at ~14,000 cells/cm^2^ in a 6-well plate for RNA extractions. Cells were treated with either a DMSO vehicle control (J66650.AD, Thermo Scientific Alfa Aesar, Haverhill, MA, USA), or a 10 µM dose of diethylstilboestrol, ethynyl estradiol, or levonorgestrel (Catalogue numbers HY-14598, HY-B0216 or HY-B0257, respectively, MedChemExpress, Stockholm, Sweden). Fresh medium was added to the plates before the addition of the treatment stock. Cells were treated for 24 h before the removal of treatment, followed by immediate staining or harvesting. 

#### 2.5.2. Quantification of Senescent Cell Load Using SAB Staining

Cultures were stained for SAB activity using the Senescence Cells Histochemical Staining kit (CS0030, Merck, Darmstadt, Germany) according to the manufacturer’s instructions. After 24 h of staining, cells were imaged at 10× magnification using a Zeiss AxioCam ERC55 PrimoVert. Five images per biological replicate were captured and later counted manually using ImageJ 1.47v software (US National Institute of Health, Bethesda, MD, USA) [26]. Differences in SAB staining between test compounds and controls were assessed by one-way ANOVA with an uncorrected Fisher’s LSD *post hoc* test and graphed using GraphPad Prism version 9.4.1 for Windows (GraphPad Software, San Diego, CA, USA, www.graphpad.com).

#### 2.5.3. Quantification of Cellular Proliferation and DNA Damage Repair Using Immunocytochemical Staining for Ki67 and γH2AX

Following two washes in DPBS (14190136, Gibco™), the cells were fixed with 4% paraformaldehyde and stored in DPBS. Prior to staining, the cells were washed again in DPBS and blocked with ADST [antibody diluent solution—triton: DPBS, 0.1 M L-Lysine (303341000, Thermo Scientific™, Waltham, MA, USA), 1% *w*/*v* Human Serum Albumin Fraction V (12668-10GM, Sigma-Aldrich, St. Louis, MO, USA), Triton X-100 (A16046.AP, Thermo Scientific Alfa Aesar)] and 5% human serum (H3667, Sigma-Aldrich) for 30 min. Antibodies were commercially derived from Abcam (Cambridge, UK): Rb anti-Ki67 (ab15580, ab16667), Ms anti-γH2AX (ab26350), Alexa Fluor ^®^ 555 Goat pAb to Rb (ab150078, ab150086) and Alexa Fluor ^®^ 488 Goat pAb to Ms (ab150117). Primary antibodies were applied overnight at 2.5 µg/mL (suspended in ADST with 2% human serum). Secondary antibodies were applied at 5 µg/mL and 4′,6-diamidino-2-phenylindole (DAPI, D1306, Invitrogen™, Waltham, MA, USA) at 1 µg/mL (suspended in ADST with 2% human serum) were applied for 1 h. Then, the coverslips were mounted with Dako mounting medium (S302380-2, Agilent, Santa Clara, CA, USA). Five representative images per coverslip were captured at 10× magnification using a Leica DM4 B Upright Microscope and cells were manually scored positive or negative for each parameter manually using the Leica Application Suite X 2019 3.7.1.21655v software (Leica Microsystems, Wetzlar, Germany). Differences in cell kinetic parameters between treated and control cells were assessed by one-way ANOVA with an uncorrected Fisher’s LSD *post hoc* test and graphed using GraphPad Prism version 9.4.1 for Windows (GraphPad Software, San Diego, CA, USA, www.graphpad.com).

#### 2.5.4. Quantification of Apoptosis Using TUNEL Assay

Cells were washed in DPBS (14190136, Gibco™), before the cells were fixed with 4% paraformaldehyde, washed again and stored in DPBS. The Click-iT^®^ TUNEL Alexa Fluor^®^ Imaging Assay (C10245, ThermoFisher) was performed according to the manufacturer’s instructions using additional DPBS, bovine serum albumin (BSA) fraction V fatty acid-free (10775835001, Roche, Basel, Switzerland), and Triton X-100 (A16046.AP, Thermo Scientific Alfa Aesar). In the same manner as for the other immunofluorescently stained cells, the Leica DM4 B Upright Microscope at 10× magnification was used to capture five images per coverslip. The cells in the images were later counted manually using Leica Application Suite X 2019 3.7.1.21655v software (Leica Microsystems, Wetzlar, Germany). Differences in TUNEL staining between test compounds and controls were assessed by one-way ANOVA with an uncorrected Fisher’s LSD *post hoc* test and graphed using GraphPad Prism version 9.4.1 for Windows (GraphPad Software, San Diego, CA, USA, www.graphpad.com).

#### 2.5.5. Quantitative RT-qPCR Assessment of Gene Expression

RNA was extracted from cells using TRI Reagent Solution (AM9738, Invitrogen™) according to the manufacturer’s instructions, with the addition of 10 mM MgCl_2_ (AM9530G, Invitrogen™) before phase separation (to aid in RNA recovery [27]) and 1.2 µL of 15 mg/mL GlycoBlue™ Coprecipitant (AM9516, Invitrogen™) prior to washing (to aid in pellet visualisation). RNA was resuspended in 20 µL 1 × TE buffer, pH 8.0 (BP2473-500, Fisher Bioreagents) and assessed for concentration and quality using the Thermo Scientific™ Nanodrop 8000 Spectrophotometer (Thermo Fisher Scientific, Waltham, MA, USA). RNA was reverse-transcribed at 10 ng/µL in a 20 µL reaction, using the High-Capacity cDNA Reverse Transcription Kit (4368813, Applied Biosystems™, Waltham, MA, USA) according to the manufacturer’s instructions. Reverse transcription was performed on an Applied Biosystems™ Veriti™ 96-Well Fast Thermal Cycler with the following cycling conditions: 25 °C for 10 min, 37 °C for 120 min, 85 °C for 5 min and a 4 °C hold step. A total of 12.5 ng of cDNA was pre-amplified according to the manufacturer’s instructions using TaqMan™ PreAmp Master Mix (4384266, Applied Biosystems™, Waltham, MA, USA) and pooled TaqMan™ Gene Expression Assays (FAM) (4331182, TaqMan^®^, Life Technologies, Carlsbad, CA, USA). Transcripts encoding factors associated with apoptosis, the senescence-associated secretory phenotype (SASP) and regulators of alternative splicing were assessed by RT-qPCR. A table of genes assessed is provided in Table 1. Using the Applied Biosystems™ Veriti™ 96-Well Fast Thermal Cycler, the cycling conditions were: 95 °C for 10 min, 14 cycles of [95 °C for 15 s, 60 °C for 4 min], 99 °C for 10 min, and a 4 °C hold step. The pre-amplified cDNA products were diluted by a factor of 10 in 1 × TE buffer, pH 8.0 (BP2473-500, Fisher Bioreagents, Thermo Fisher Scientific, Waltham, MA, USA). RT-qPCR was performed in three biological and three technical replicates on the Quantstudio 12K platform (Applied Biosystems™, Waltham, MA, USA) as 5 µL reactions on 384-well plates. The cycling conditions were: 50 °C for 2 min, 95 °C for 10 min, followed by 50 cycles of 95 °C for 15 s and 60 °C for 1 min. 1 µL of diluted, pre-amplified cDNA product was used per reaction with 0.25 µL of Taqman™ Gene Expression Assay (equating to 900 nM primer and 250 nM probe). Gene expression was calculated using the comparative C_T_ technique [25] relative to the geometric mean of five housekeeping genes (*GUSB*, *IDH3B*, *PGK1*, *PPIA* and *UBC*) empirically selected for stability as described above [24], and normalized to expression levels in the respective cell type’s vehicle-treated control. Results were assessed for statistical significance using a one-way ANOVA with an uncorrected Fisher’s LSD *post hoc* test and graphed using GraphPad Prism version 9.4.1 for Windows (GraphPad Software, San Diego, CA, USA, www.graphpad.com).

### 2.6. Bioinformatic Assessment of Structure–Function Relationships

Structural information on each compound tested was obtained from the supplier (MedChemTronica, Stockholm, Sweden). The SMILES (simplified molecular input line entry system) data was transformed into SDF (structure data file) data for analysis with ChemmineR and fmcsR packages in Rstudio software version 4.1.0 [28,29,30,31]. Tanimoto coefficients, measures of structural similarity [28], were computed for each pair of compounds and used to construct a matrix of intragroup comparisons of structural similarity. Computing resource limitations imposed a maximum of 30 compounds per test group. The average Tanimoto coefficient across the matrix of functionally related compounds was compared against the average Tanimoto coefficient for a control group of non-functionally related compounds using an unpaired t test in GraphPad Prism version 9.4.1 for Windows (GraphPad Software, San Diego, CA, USA, www.graphpad.com). A dendrogram was constructed for groups of interest to illustrate the structural similarity between compounds. If a structure–function relationship was suggested, the exact maximum common substructure was computed for the two least similar compounds (as identified in the dendrogram) to identify the maximum common substructure across the whole test group.

#### 2.6.1. Methodological Validation

Producing a statistical comparison between similarity matrices represents a novel use for the matrix outputs of ChemmineR. A significant difference between intragroup average Tanimoto coefficients indicates that compounds in the test group are more structurally similar than the control group. When the test group contains only compounds with a particular function of interest, a significant difference may suggest a structure–function relationship. The maximum common substructure of the group may therefore suggest (or be incorporated within) a substructure which is associated with the function of interest. To validate this approach, a group of compounds that share a known functionally related substructure was compared against a control group. Validation compounds were selected from the MedChemExpress FDA-Approved Drug Library Plus panel of 2278 compounds (MedChemTronica, Stockholm, Sweden).

Given that our in vitro screens had already highlighted some oestrogenic compounds and that the provided drug library information identified compounds that target the oestrogen receptor, we decided to validate the approach by trying to identify a known structure–function relationship using compounds that target the oestrogen receptor. These compounds are known to share substructures which are linked with their function of targeting the oestrogen receptor. The validation test group consisted of 30 compounds versus a control group of 30 functionally unrelated compounds. The number of oestrogenic compounds in the validation test group was varied to assess the sensitivity of the method: using 30, 10, 4, 3 and 2 oestrogenic compounds in a group of other non-oestrogenic compounds totaling 30 for comparison against the control group of 30 functionally unrelated compounds.

#### 2.6.2. Structure–Function Analysis of In Vitro Screen Results

The first two test groups were the compounds that had either increased or decreased *CDKN2A* gene expression the most (averaged across both doses). The third test group was a selection of compounds that had decreased SAB activity in the screen. Control compounds acting as a non-functionally associated control group were selected based on the compounds with the least effect on *CDKN2A* expression and were matched to the number of compounds in each test group. In total, 78 individual compounds were used in the study. Test groups of compounds are described in Supplementary Figures S2–S4.

## 3. Results

### 3.1. Primary and Secondary Screens

**Figure 2:**
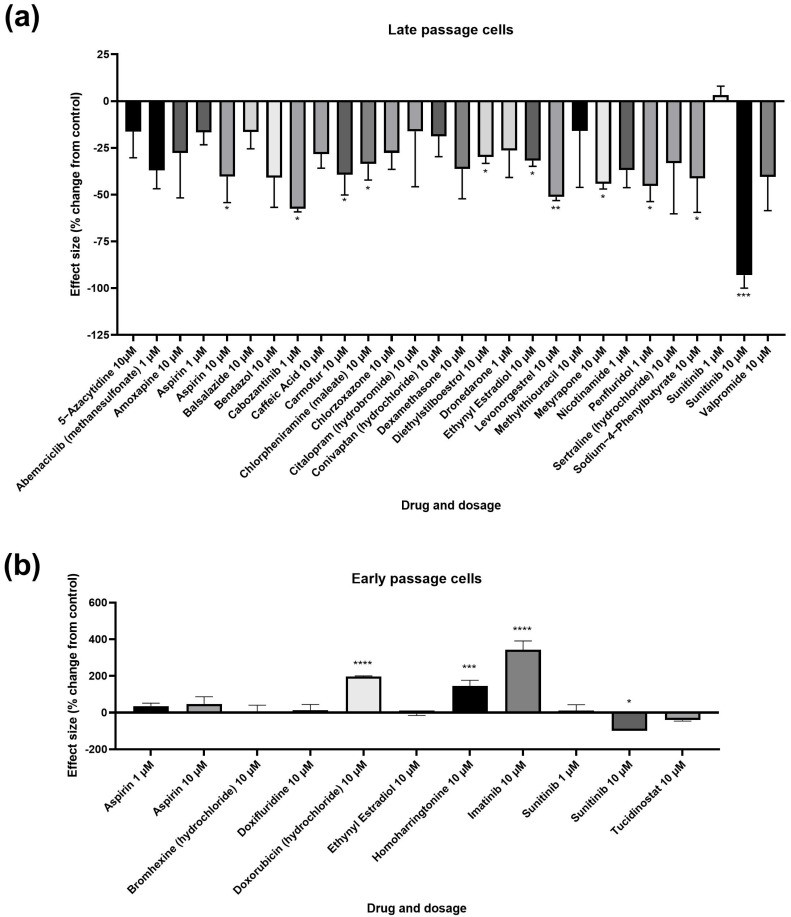
Assessment of effects on senescent cell load using senescence-associated beta galactosidase (SAB) activity. (**a**). Effect of treatments on SAB activity in cells at a late passage with higher levels of SAB activity. (**b**). Effect of treatments on SAB activity in cells at an early passage with low levels of SAB activity. Error bars show standard error of the mean (SEM), and statistical significance of *p* values computed using one-way ANOVA with uncorrected Fisher’s LSD *post hoc* tests. * *p* < 0.05, ** *p* < 0.01, *** *p* < 0.001 and **** *p* < 0.0001.

We identified 90 molecules that altered *CDKN2A* gene expression in male cells, with 20 increasing senescent cell load and 70 decreasing senescent cell load by more than mean ± three standard deviations of the control treatments (Table 2). 32 compounds were selected for secondary screening based on effect size, widespread usage or due to having different effect directionality between doses from the primary screen. Of these, 11 compounds elicited a reduction in SAB positivity, and three caused an increase in SAB positivity (Figure 2; Table 3). Compounds causing a statistically significant decrease in SAB activity included the non-steroidal anti-inflammatory drug (NSAID) aspirin, the cancer drugs cabozantinib, and carmofur, the antihistamine chlorpheniramine (maleate), the 11β-hydroxylase inhibitor metyrapone, the antipsychotic penfluridol, the ammonia lowering drug sodium-4-phenylbutyrate and the synthetic female sex hormones diethylstilboestrol, ethynyl estradiol and levonorgestrel. Most of these effects were evident at 10 µM concentration, though aspirin and penfluridol had effects at a lower concentration of 1 µM. Although the cancer drug, sunitinib, caused a significant decrease in SAB activity at 10 µM in both early and late passage cells, the drug caused mass cell death rather than acting as a senotherapeutic. Compounds demonstrating induction of senescence included the anticancer agents: doxorubicin, homoharringtonine and imatinib. 

### 3.2. Potential Donor Characteristic-Specific Differences in Cellular Senescence Kinetics in Response to Treatment with Female Synthetic Sex Hormones

**Figure 3:**
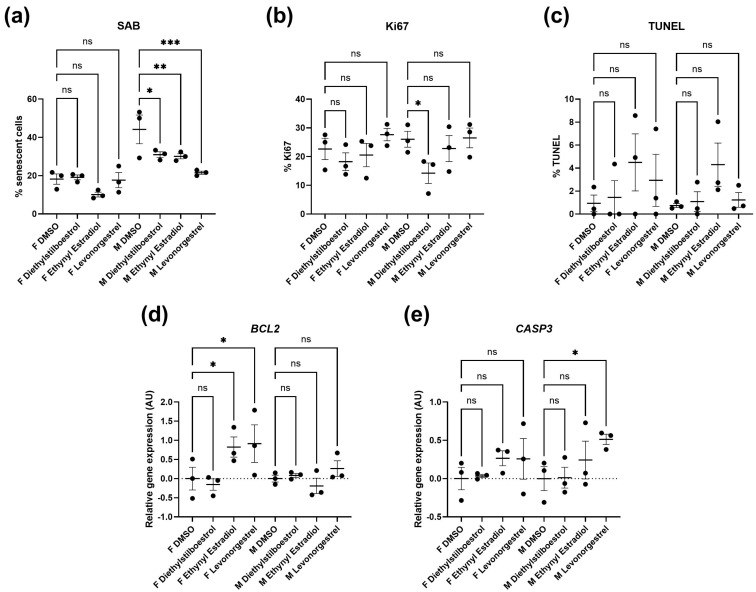
Senescence kinetics for senescent male and female primary dermal fibroblasts. Percentage of cells stained for (**a**). senescence-associated beta galactosidase (SAB), (**b**). Ki67, a marker of proliferation and (**c**). Terminal deoxynucleotidyl transferase dUTP nick end labeling (TUNEL), a marker of DNA damage, in female (F) and male (M) dermal fibroblast cells treated with synthetic female hormones at 10 µM or a DMSO-only control. Gene expression of markers for apoptosis, (**d**). *BCL2* and (**e**). *CASP3*, in female (F) and male (M) dermal fibroblast cells. *n* = 3 for all groups. Error bars show standard error of the mean (SEM), and statistical significance of *p* values computed using one-way ANOVA with uncorrected Fisher’s LSD *post hoc* tests is reported: (ns) not significant, * *p* < 0.05, ** *p* < 0.01 and *** *p* < 0.001.

**Figure 4:**
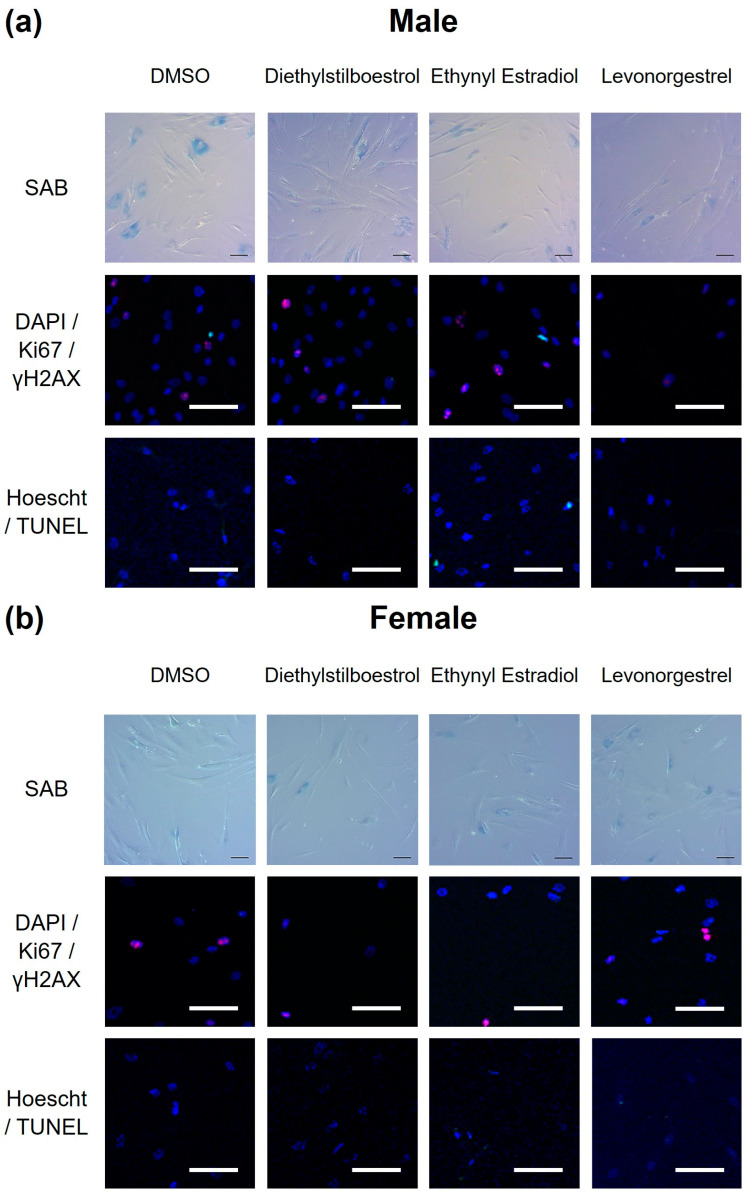
Example of staining for senescence kinetics for senescent male (**a**) and female (**b**) primary dermal fibroblasts. Cells are stained for senescence-associated beta galactosidase (SAB; a marker of senescence), Ki67 (a marker of proliferation), γH2AX (a marker of DNA damage repair), and Terminal deoxynucleotidyl transferase dUTP nick end labelling (TUNEL; a marker of DNA damage) in female and male dermal fibroblast cells treated with synthetic female hormones at 10 µM or a DMSO-only control. SAB staining appears blue and was imaged using brightfield microscopy. Cells were multiplex stained for Ki67 (red) and γH2AX (green) with nuclei stained for DAPI (blue), and imaged on a fluorescence microscope at 10× magnification. Cells were stained for TUNEL (green) and Hoescht (blue), and imaged on a fluorescence microscope at 10× magnification. Scale bars indicate 100 µm.

Female synthetic sex hormones were prominent across both screens, so we examined the effects of these compounds in more detail in senescent male and female primary dermal fibroblasts. We identified that all three synthetic female hormones caused a decrease in SAB activity in male fibroblasts (a 30%, 32% and 51% decrease in stained cells for diethylstilboestrol, ethynyl estradiol and levonorgestrel; *p* = 0.0122, 0.0083 and 0.0002, respectively). Notably, these effects were not evident in the female cells (Figure 3a and Figure 4; Supplementary Table S2).

Effects on proliferation were minimal, with only diethylstilboestrol demonstrating a 45% decrease in proliferation (*p* = 0.0289) in male cells (Figure 3b and Figure 4; Supplementary Table S2). Levels of γH2AX, (indicating DNA damage repair) were very low in all cell types and treatments, reflected in very low levels of cell death in the culture as measured by TUNEL assay; an average of 2.1% of cells had evidence of double strand breaks with no significant difference noted between any of the experimental groups (Figure 3c and Figure 4; Supplementary Table S2). We also noted some donor-specific changes in apoptotic markers (Figure 3; Supplementary Tables S3 and S4). We observed an 82% and a 91% increase in *BCL2* expression following treatment with ethynyl estradiol or levonorgestrel in female cells (*p* = 0.0351 and 0.0214). *BCL2* expression was unchanged in male cells. Conversely, *CASP3* was increased by 51% in response to levonorgestrel (*p* = 0.0397) in male cells but was unchanged in female cells.

**Figure 5:**
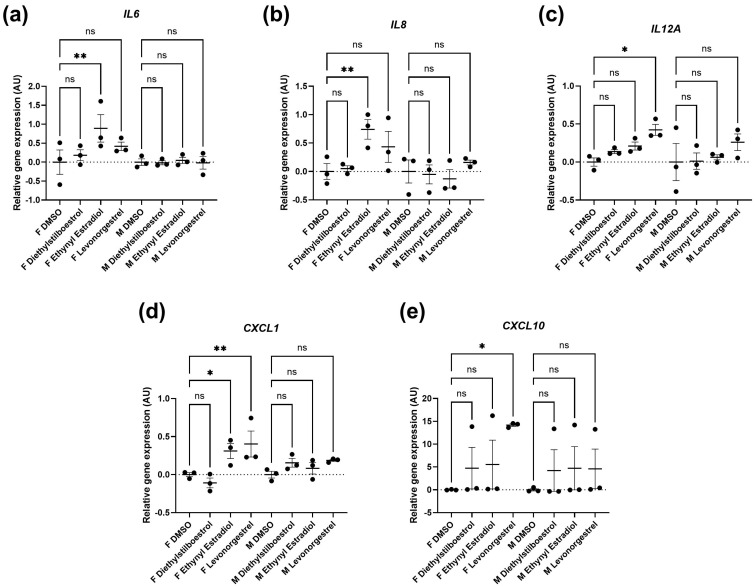
Gene expression of genes encoding senescence-associated secretory phenotype (SASP) factors in female (F) and male (M) dermal fibroblast cells treated with synthetic female hormones at 10 µM or a DMSO-only control. Graph demonstrating the effect of synthetic female sex hormones on (**a**). *IL6*, (**b**). *IL8*, (**c**). *IL12A*, (**d**). *CXCL1* and (**e**). *CXCL10* expression. Error bars show standard error of the mean (SEM), and statistical significance of *p* values computed using one-way ANOVA with uncorrected Fisher’s LSD *post hoc* tests is reported: (ns) not significant, * *p* < 0.05 and ** *p* < 0.01.

Furthermore, the treatments only affected SASP factor expression in the female cells, appearing to be mildly pro-inflammatory (Figure 5; Supplementary Tables S3 and S4). Diethylstilboestrol caused an 89% increase in *IL6* expression (*p* = 0.0057) and increased *IL8* expression by 74% (*p* = 0.0062). Ethynyl estradiol caused a 54% increase in expression of *CXCL1* (*p* = 0.0174). Levonorgestrel caused a very large effect in *CXCL10* expression (a 14-fold increase, *p* = 0.0162) but it is important to note that gene expression of *CXCL10* in the controls was very low. Levonorgestrel also elicited a 40% and an 42% increase in *CXCL1* and *IL12A* expression, respectively (*p* = 0.0033 and 0.0148) No other SASP markers were altered in either the male or the female cells.

**Figure 6:**
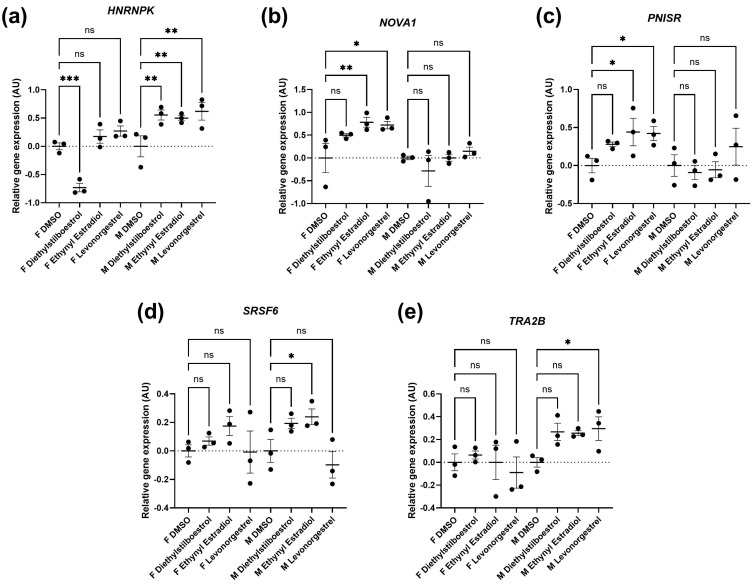
Splicing factor expression following treatment with synthetic female sex hormones. Graph demonstrating the effect of synthetic female sex hormones on (**a**). *HNRNPK*, (**b**). *NOVA1*, (**c**). *PNISR*, (**d**). *SRSF6* and (**e**). *TRA2B* expression. *n* = 3 for all groups. Error bars show standard error of the mean (SEM), and statistical significance of *p* values computed using one-way ANOVA with uncorrected Fisher’s LSD *post hoc* tests is reported: (ns) not significant, * *p* < 0.05, ** *p* < 0.01 and *** *p* < 0.001.

Splicing factor dysregulation is known to be a driver of senescence, and targeted restoration of splicing factor expression yields senomorphic effects [32]. We noted differences in the expression of splicing factor genes between the male and the female primary dermal fibroblasts in response to synthetic female sex hormones (Figure 6; Supplementary Tables S3 and S4). Diethylstilboestrol, ethynyl estradiol, and levonorgestrel induced a 55%, 50% and 62% increase in *HNRNPK* expression in male cells (*p* = 0.0029, 0.0060 and 0.0012, respectively), whereas diethylstilboestrol caused a 73% decrease in female cells (*p* = 0.0003). In male cells, ethynyl estradiol induced a 24% increase in *SRSF6* expression (*p* = 0.0449) and levonorgestrel induced a 30% increase in *TRA2B* expression (*p* = 0.0361), but no effects on expression of either gene were observed in female cells. In female cells, ethynyl estradiol and levonorgestrel induced a 78% and 72% increase in the gene expression of the spliceosomal component, *NOVA1* (*p* = 0.0065 and 0.0109, respectively), and induced a 44% and 42% increase in *PNISR* expression (*p* = 0.0372 and 0.0452, respectively), whereas male cells were unaffected. 

### 3.3. A Common Substructure Was Identified for Compounds That Decreased CDKN2A

A structure–function analysis was used to identify any substructure associated with compounds that were grouped by their functionality from the screens for *CDKN2A* gene expression and/or SAB activity. Prior to this analysis, methodological validation of the bioinformatic statistical approach indicated that the method is not very sensitive. Only the methodological validation test group consisting of 30 oestrogen receptor-targeting compounds (mean Tanimoto coefficient ± standard error of the mean (SEM); 0.3634 ± 0.007869, *n* = 900) versus 30 control compounds (0.1886 ± 0.005936, *n* = 900) was significantly different (*p* < 0.0001). Variations of the 30-compound validation test group with fewer oestrogenic compounds and more functionally unrelated compounds were not significant when compared against the control group: ten oestrogenic compounds (0.1877 ± 0.006082, *n* = 900, *p* = 0.9167), five oestrogenic compounds (0.1820 ± 0.006138, *n* = 900, *p* = 0.4418), three oestrogenic compounds (0.1885 ± 0.006182, *n* = 900, *p* = 0.9985), two oestrogenic compounds (0.1861 ± 0.006148, *n* = 900, *p* = 0.7690), and a group with zero oestrogenic compounds (0.1851 ± 0.006323, *n* = 900, *p* = 0.6895).

**Figure 7:**
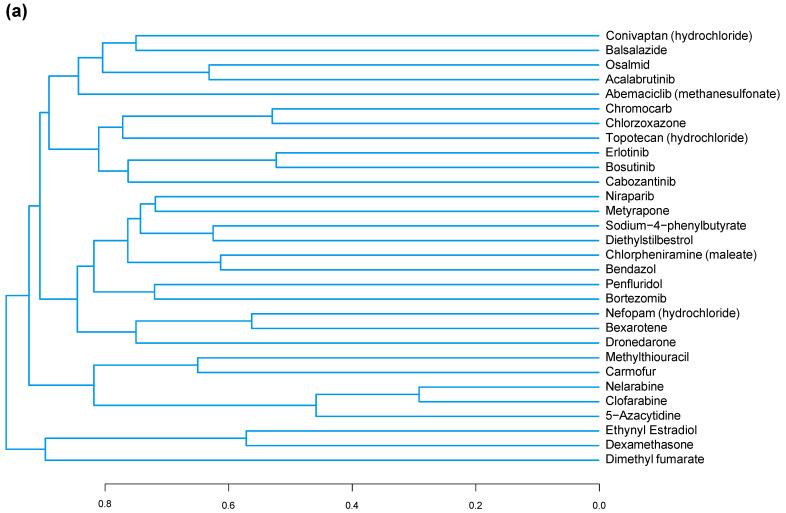

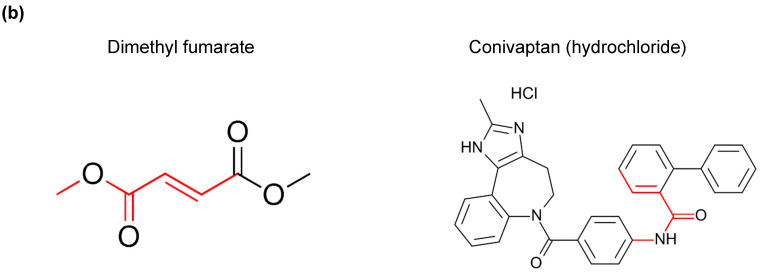
Structure-function analysis of compounds that decreased *CDKN2A* expression. (**a**). Dendrogram constructed using the Tanimoto coefficient to show structural similarity of compounds tested that decreased *CDKN2A* gene expression. (**b**). Maximum common substructure of the two least structurally similar compounds that decreased *CDKN2A* gene expression.

In total, 78 individual compounds (and the associated data on their effects on *CDKN2A* expression and/or SAB activity) were used to provide input for a structure–function analysis (Supplementary Figures S2–S4). The first test group consisted of the 30 compounds that decreased *CDKN2A* expression the most (averaged across both doses). Structures for this group are shown in Supplementary Figure S2. This test group (0.2107 ± 0.005580, *n* = 900) was significantly structurally different from the control group (0.1755 ± 0.006050, *n* = 900) with a *p* < 0.0001. A dendrogram was constructed to visualize the structural similarity of the compounds in the group in Figure 7a. The maximum common substructure between the two least similar compounds in the second test group is shown in red in Figure 7b.

The number of compounds that increased SAB activity (*n* = 3) was too small to be suitable for this type of analysis, but the number of compounds that decreased SAB activity was appropriate (*n* = 11). Sunitinib was omitted from the analysis as it caused mass cell death rather than acting to reduce senescence. Test group two therefore consisted of a group of ten compounds that decreased SAB activity (Supplementary Figure S3), and their Tanimoto coefficients were compared against the coefficients of a control group of ten compounds that did not have an effect on *CDKN2A* expression. The average Tanimoto coefficient was not significantly different in the test group (0.2928 ± 0.02544, *n* = 100) compared to the control group (0.2524 ± 0.02900, *n* = 100, *p* = 0.2964).

The third test group (Supplementary Figure S3) comprised the eight compounds that increased *CDKN2A* expression (above the mean ± 3 SDs criterion) when averaged across both doses. The average Tanimoto coefficient of this group (0.2817 ± 0.03492: mean ± SEM, *n* = 64) had no significant difference when compared against the average Tanimoto coefficient of eight compounds that had no effect on *CDKN2A* expression (0.2482 ± 0.03798, *n* = 64, *p* = 0.5169).

## 4. Discussion

We performed a drug repurposing screen on 240 FDA-approved molecules for effects on cellular senescence phenotypes. We identified 90 compounds that have effects on *CDKN2A* expression in human primary dermal fibroblasts, 11 of which bring about a reduction in senescent cell load and 3 of which increase senescent cell load as measured by SAB activity. Three of the compounds that reduce senescent cell load are the synthetic female sex hormones diethylstilboestrol, ethynyl estradiol and levonorgestrel, which exert senotherapeutic effects in male dermal fibroblasts, but not in female cells, where their effects are mildly inflammatory. Finally, we have identified a chemical substructure associated with reduced *CDKN2A* expression. Our findings are important for future research into drugs to target the molecular basis of ageing, as they indicate that some senotherapeutic effects may be specific to certain donor characteristics, e.g., sex, which has major implications for therapeutic screening cascades and eventual population level treatment options. 

Several of the compounds that we identified as having effects on senescence phenotypes are frequently prescribed or are common household drugs, some of which have also been previously linked with pathways associated with ageing. Aspirin, for example has been shown to extend lifespan in mice [33], metyrapone is an 11β-hydroxylase inhibitor known to activate autophagy [34,35] and penfluridol, a potent antipsychotic medication, has been shown to increase lifespan in *Drosophila melanogaster* [36]. Several known senotherapeutic compounds (dasatinib (hydrochloride), metformin (hydrochloride), resveratrol and trametinib) were not amongst the largest effects on *CDKN2A*, suggesting that smaller effects could also be worth examining in similar screens in the future. In the primary screen, we observed that some drug classes had interesting effects on senescence, suggesting that more research is needed into these effects as it may be that certain drugs are more (or less) suitable for use in older patients due to their effects on senescence. Unsurprisingly, we detected effects on senescence kinetics for drugs used in the treatment of cancer, but perhaps less predictably, we also detected effects for antidepressant drugs, anticonvulsant drugs, and female synthetic sex hormones. 

Our study has identified a maximum common structural motif that was present even in molecules with very little other structural similarity. This compares well with work in the literature from Olascoaga-Del Angel et al., where several chemotypes associated with senomorphic or senolytic properties were identified [37]. The maximum common substructure that we identified was also common across 11 of the 13 structures in their larger-scale analysis [37]. This finding is strengthened when we consider that the new approach used for the identification of structural similarities was not very sensitive, as noted during the methodology validation.

Diethylstilboestrol, ethynyl estradiol and levonorgestrel were all associated with a decrease in *CDKN2A* expression in male cells. These drugs are commonly used in hormone replacement therapy, contraceptives or as oncodrugs [38,39]. Female hormones are associated with protective benefits in ageing [40,41,42], and there is some evidence of sex differences in senescence-associated phenotypes [43,44,45,46,47,48,49,50,51,52,53]. It is clear that being biologically female offers protective benefits against ageing [46,54], and the two main female hormones, oestrogen and progesterone, are known to be involved in many ageing and senescence-related pathways [47,55,56,57,58]. The typical nuclear receptors for these two hormones, the oestrogen receptors (ERα and ERβ) and progesterone receptors (PR-A and PR-B) are involved in the same pathways [57,59]. There is comparatively little information about the senotherapeutic properties of synthetic female sex hormones in humans [48,60,61]; most research has been carried out in mouse models treated with synthetic oestrogens [58,62,63,64].

We found differences in SAB positivity, expression of splicing factors and expression of mRNAs encoding SASP proteins between male and female cells in response to female sex hormones. Sex differences in drug responses are not uncommon, and a sexual dimorphism has been reported in mice in response to senotherapeutics [65,66]. Recently, the NIA Interventions Testing Program in mice has revealed sex differences in effects on longevity in response to 17-α-estradiol and aspirin [67,68]. Anthropometric parameters such as bodyweight, fat distribution, and differences in pharmacokinetics and pharmacodynamics may mean that women are more sensitive to some drugs, have altered clearance kinetics and may experience more drug interactions [69]. In humans, oestrogen and progesterone are endogenous to both sexes, but differ in their circulating levels [70,71]. Unlike progesterone, there are many forms of oestrogen: estrone (E1), estradiol (E2), estriol (E3), and other minor oestrogens, but the major oestrogen is E2. This has two isoforms: 17α-estradiol and the more potent and biologically-most relevant 17β-estradiol [59,72]. Oestrogens are discussed more often than progesterones in relation to senescence, but in this study levonorgestrel, a progesterone, had a larger effect on senescence than the oestrogens. Diethylstilboestrol decreased proliferation in male cells, which is at odds with oestrogen’s often growth-inducing effects, e.g., during the female pubertal growth spurt [70]. Ethynyl estradiol caused an increase in *BCL2* expression in female cells only, but did not affect other markers of apoptosis or DNA damage. At this time, it is not clear whether the observed sex differences arise from differences in bioavailability, or from an undescribed non-canonical role of the hormones over and above canonical oestrogen/progesterone signaling, particularly given the senomorphic effect occurs with treatment of either a synthetic oestrogen or a progesterone. The classical signaling pathways for both oestrogen and progesterone feature the hormone and its respective nuclear receptor(s) acting as ligand-activated transcription factors. The complex binds to hormone responsive elements (HREs) in the genome to control gene expression. There are many HREs across the genome, for example there are over 70,000 oestrogen-responsive-elements identified [73]. Both hormones can act via other pathways, including membrane bound GPCRs. Activation of their respective GPCRs can activate cell fate pathways such as Ras/Raf/MEK/ERK and PI3K/Akt, as well as cross-signaling with classical hormonal signaling pathways [74,75]. Both pathways have previously been implicated in senescence in human cells [21] and in lifespan in invertebrate models [76]. Differing expression, activity and/or sensitivity of receptors between the sexes might also be factoring into the senotherapeutic effect observed in this study. Another consideration is that the female fibroblasts used in our study were donated by a pre-menopausal woman: it is possible that cells from women who are undergoing or have gone through the menopause may have differing responses to synthetic female hormones, or indeed they may have a similar effect compared to the effect seen in the male cells. 

Translating the findings of repurposing screens into the clinic needs careful consideration. When considering these compounds in vivo, dosage is also a factor. Many compounds associated with senomorphic effect display biphasic dose responses, which may arise from the autoregulatory relationships between the affected genes and pathways [21]. It is therefore possible that repeated exposure and/or higher/lower dosage may have different effects in a systemic setting. It is also possible that the effects may be tissue specific and/or donor-specific: future studies should use multiple cell types from multiple donors of different sexes. Repurposing drugs identified to have senotherapeutic effect may also not be clinically feasible as severe side effects may alter the risk-benefit relationship for milder age-related diseases. The three female synthetic hormones identified in this study do not currently offer a potential clinical application as a mainstream senotherapeutic drug as the effect is not observed in females who routinely take the medicines, and males taking the hormones may have feminising side-effects. Indeed, diethylstilboestrol has been linked with transgenerational cancers and may not be appropriate in the context of senotherapeutics.

## 5. Conclusions

Our work demonstrates the utility of repurposing screens, combined with bioinformatic structure–function analyses to identify chemical structures that may be suitable for eventual senotherapeutic benefit. Our study suggests that the sexual dimorphisms in senomorphic/geroprotective effects in animal models may also exist in human cells. We identify several compounds of interest for future senotherapeutic research in the screen including the three female synthetic hormones. We use a new approach to also identify a chemical substructure associated with a decrease in senescence. Our work also highlights the need for patient characteristics such as biological sex to be taken into consideration even in early in vitro pre-clinical work; high throughput screening cascades are often carried out using a single clone of a well characterized transformed cell line, and other senotherapeutic compounds may be sex-specific. This statement could equally be applied to other individual anthropometric or genetic characteristics. Biological sex in in vitro experiments can cause dimorphic effects and this should be considered more regularly when designing experiments, particularly in the process of investigating senotherapeutic compounds. The easiest cell type may not always be the best candidate for such screens. However, provided that studies are designed appropriately to factor in donor characteristics such as sex, repurposing remains a potent mechanism for identifying new jobs for old drugs.

## Figures and Tables

**Table 1:** Gene name and TaqMan™ Gene Expression assay IDs used for characterization experiments.

Gene Name	Assay ID	Gene Name	Assay ID
*AKAP17A*	Hs00946624_m1	*IL-10*	Hs00961622_m1
*ATM*	Hs00175892_m1	*IL12A*	Hs01073447_m1
*BCL2*	Hs04986394_s1	*IL12B*	Hs01011518_m1
*CASP1*	Hs00354836_m1	*IL-1B*	Hs01555410_m1
*CASP3*	Hs00234387_m1	*IL-2*	Hs00174114_m1
*CASP7*	Hs00169152_m1	*IL-6*	Hs00174131_m1
*CASP8*	Hs06630780_s1	*INFγ*	Hs00989291_m1
*CASP9*	Hs00962278_m1	*LTA (TNFβ)*	Hs99999086_m1
*CXCL1*	Hs00236937_m1	*MMP1*	Hs00899658_m1
*CXCL10*	Hs00171042_m1	*MMP3*	Hs00968305_m1
*CXCL8 (IL-8)*	Hs00174103_m1	*MMP9*	Hs00957562_m1
*GUSB*	Hs00939627_m1	*NOVA1*	Hs00359592_m1
*HNRNPA0*	Hs00246543_s1	*PGK1*	HS99999906_m1
*HNRNPA1*	Hs01656228_s1	*PNISR*	Hs00369090_m1
*HNRNPA2B1*	Hs00242600_m1	*PPIA*	Hs04194521_s1
*HNRNPD*	Hs01086912_m1	*SRSF1*	Hs00199471_m1
*HNRNPH3*	Hs01032113_g1	*SRSF2*	Hs00427515_g1
*HNRNPK*	Hs00829140_s1	*SRSF3*	Hs00751507_s1
*HNRNPM*	Hs00246018_m1	*SRSF6*	Hs00607200_g1
*HNRNPUL2*	Hs00859848_m1	*SRSF7*	Hs00196708_m1
*IDH3B*	Hs00199382_m1	*TNFα*	Hs00174128_m1
*IL-10*	Hs00961622_m1	*TRA2β*	Hs00907493_m1
*IL12A*	Hs01073447_m1	*UBC*	Hs01871556_s1

**Table 2:** Fold change in *CDKN2A* (arbitrary units, relative to control) by compound and dose in the initial senescence screen. All effects listed here were more than three standard deviations above or below the mean of control treatments.

Drug Name	Dose (µM)	Fold Change in *CDKN2A*
Tucidinostat	10	2.048
Doxifluridine	10	1.559
Doxorubicin (hydrochloride)	10	1.498
Bromhexine (hydrochloride)	10	1.167
Homoharringtonine	10	1.160
Chlorambucil	10	1.133
Aspirin	10	1.072
Amoxapine	10	1.034
Doxorubicin (hydrochloride)	1	0.969
Imatinib	10	0.948
Montelukast (sodium)	10	0.888
Atorvastatin (hemicalcium salt)	10	0.822
Ribociclib	10	0.820
Baricitinib (phosphate)	10	0.820
Irinotecan (hydrochloride)	10	0.804
Levoleucovorin (calcium)	10	0.798
Epirubicin (hydrochloride)	10	0.790
Cobimetinib	10	0.773
Homoharringtonine	1	0.765
Decitabine	10	0.744
Sunitinib	10	0.722
Temozolomide	10	0.700
Silibinin	10	−0.686
Diacerein	10	−0.694
Vinorelbine (ditartrate)	1	−0.713
Alpelisib	10	−0.717
Ethamsylate	10	−0.734
Diethylstilboestrol	1	−0.753
Altretamine	10	−0.782
Panobinostat	1	−0.791
Sertraline (hydrochloride)	1	−0.805
Deferoxamine (mesylate)	10	−0.822
Balsalazide	1	−0.852
Pexidartinib	1	−0.890
Bexarotene	10	−0.894
Clofarabine	10	−0.897
Caffeic acid	10	−0.903
Pazopanib (hydrochloride)	10	−0.909
Aspirin	1	−0.916
Dexamethasone	1	−0.917
Pazopanib	10	−0.921
Rucaparib (phosphate)	10	−0.984
Glasdegib	1	−1.005
Aceglutamide	10	−1.020
Trimethoprim	10	−1.021
Crizotinib (hydrochloride)	10	−1.051
Acalabrutinib	1	−1.069
Zidovudine	10	−1.080
Citalopram (hydrobromide)	10	−1.094
Topotecan (hydrochloride)	10	−1.111
Rucaparib (phosphate)	1	−1.126
Alpelisib	1	−1.153
Sertraline (hydrochloride)	10	−1.154
Erlotinib	1	−1.157
Triclabendazole	10	−1.168
Nefopam (hydrochloride)	10	−1.174
Altretamine	1	−1.184
Bortezomib	1	−1.212
Nefopam (hydrochloride)	1	−1.217
Penfluridol	10	−1.230
Clioquinol	10	−1.241
Ethynyl estradiol	1	−1.259
Panobinostat	10	−1.260
Clofibrate	1	−1.272
Mizoribine	10	−1.291
Belinostat	10	−1.330
Valpromide	10	−1.351
Bosutinib	1	−1.354
Berberine (chloride hydrate)	10	−1.367
Nelarabine	1	−1.403
Acalabrutinib	10	−1.405
Tofacitinib (citrate)	10	−1.412
Erdosteine	1	−1.470
Bortezomib	10	−1.475
Bosutinib	10	−1.478
Osalmid	1	−1.493
Topotecan (hydrochloride)	1	−1.515
Bezafibrate	10	−1.523
Orotic acid	10	−1.532
Methylthiouracil	1	−1.551
Chlorpheniramine (maleate)	10	−1.559
Nitisinone	1	−1.561
Teniposide	10	−1.577
Sulfasalazine	10	−1.584
Pemetrexed (disodium hemipenta hydrate)	1	−1.702
Nifuroxazide	10	−1.705
Osalmid	10	−1.716
Nicotinamide	1	−1.717
Erlotinib	10	−1.741
Bendazol	1	−1.820
Bexarotene	1	−1.835
5-Azacytidine	1	−1.837
Nelarabine	10	−1.893
Clofarabine	1	−1.905
Niraparib	10	−1.927
Mycophenolic acid	10	−1.963
5-Azacytidine	10	−2.022
Chlorzoxazone	1	−2.045
Metyrapone	1	−2.066
Dimethyl fumarate	10	−2.099
Dexamethasone	10	−2.209
Dimethyl fumarate	1	−2.227
Chromocarb	10	−2.277
Penfluridol	1	−2.460
Bendazol	10	−2.486
Methylthiouracil	10	−2.527
Ethynyl estradiol	10	−2.684
Abemaciclib (methanesulfonate)	10	−2.768
Conivaptan (hydrochloride)	10	−2.908
Sunitinib	1	−2.926
Diethylstilbestrol	10	−3.068
Dronedarone	1	−4.099
Sodium 4-phenylbutyrate	10	−4.861
Cabozantinib	10	−7.875
Metyrapone	10	−8.532
Abemaciclib (methanesulfonate)	1	−11.417
Cabozantinib	1	−11.571
Carmofur	10	−11.805
Balsalazide	10	−11.887
Chlorzoxazone	10	−12.035

**Table 3:** Results from a screen for senescence-associated beta galactosidase (SAB) activity in male human dermal fibroblasts. The mean percentages of cells stained for SAB were compared against the corresponding DMSO-only vehicle control for each batch of the screen. Assays 1–5 were performed on later passage fibroblasts to investigate potential reductions in senescence. Assays 6–7 were performed on earlier passage fibroblasts to investigate potential increases in senescence. The mean ± standard error of the mean (SEM) and *p* values from one-way ANOVAs with Fisher’s LSD *post hoc* test are reported: (-) not applicable, (ns) not significant, * *p* < 0.05, ** *p* < 0.01, *** *p* < 0.001 and **** *p* < 0.0001.

Treatment	Mean	SEM	*p*	Significance
Assay 1 Control 10 µM	44.17	7.506	-	-
5-Azacytidine 10 µM	36.99	6.191	0.2496	ns
Caffeic Acid 10 µM	31.67	3.34	0.0553	ns
Chlorpheniramine (maleate) 10 µM	29.33	3.805	0.0264	*
Diethylstilboestrol 10 µM	30.98	1.507	0.0445	*
Ethynyl estradiol 10 µM	30.1	1.317	0.0337	*
Levonorgestrel 10 µM	21.54	0.8541	0.002	**
Assay 2 Control 10 µM	40.05	9.082	-	-
Amoxapine 10 µM	28.92	9.597	0.4353	ns
Bendazol 10 µM	23.67	6.348	0.2568	ns
Citalopram (hydrobromide) 10 µM	33.56	11.83	0.6466	ns
Methylthiouracil 10 µM	33.69	12.09	0.6531	ns
Sertraline (hydrochloride) 10 µM	26.81	10.88	0.3556	ns
Valpromide 10 µM	23.84	7.242	0.2615	ns
Assay 3 Control 10 µM	27.52	3.686	-	-
Balsalazide 10 µM	22.99	2.48	0.3251	ns
Carmofur 10 µM	16.7	2.985	0.0288	*
Chlorzoxazone 10 µM	19.91	2.438	0.109	ns
Conivaptan (hydrochloride) 10 µM	22.33	2.988	0.2627	ns
Metyrapone 10 µM	15.36	0.7593	0.0161	*
Sodium-4-phenylbutyrate 10 µM	16.16	4.995	0.0228	*
Assay 4 Control 1 µM	19.19	4.546	-	-
Abemaciclib (methanesulfonate) 1 µM	12.09	1.888	0.1025	ns
Cabozantinib 1 µM	8.13	0.2987	0.0159	*
Dronedarone 1 µM	14.12	2.768	0.234	ns
Nicotinamide 1 µM	12.11	1.795	0.1034	ns
Penfluridol 1 µM	10.48	1.586	0.0495	*
Assay 4 Control 10 µM	16.11	4.794	-	-
Dexamethasone 10 µM	10.26	2.553	0.1728	ns
Assay 5 Control 1 µM	39.1	8.275	-	-
Aspirin 1 µM	32.61	2.587	0.3269	ns
Sunitinib 1 µM	40.42	1.816	0.8398	ns
Assay 5 Control 10 µM	36.06	3.345	-	-
Aspirin 10 µM	21.39	4.997	0.0396	*
Sunitinib 10 µM	2.563	2.563	0.0002	***
Assay 6 Control 1 µM	3.163	0.5069	-	-
Aspirin 1 µM	4.287	0.5053	0.4963	ns
Sunitinib 1 µM	3.493	1.016	0.2333	ns
Assay 6 Control 10 µM	4.223	0.6868	-	-
Aspirin 10 µM	6.227	1.665	0.8404	ns
Sunitinib 10 µM	0	0	0.0199	*
Imatinib 10 µM	18.67	2.064	<0.0001	****
Assay 7 Control 10 µM	4.07	0.8632	-	-
Bromhexine (hydrochloride) 10 µM	5.65	1.818	0.3679	ns
Doxifluridine 10 µM	6.04	1.637	0.2654	ns
Doxorubicin (hydrochloride) 10 µM	15.84	0.1804	<0.0001	****
Ethynyl estradiol 10 µM	5.33	0.8184	0.4704	ns
Homoharringtonine 10 µM	13.11	1.609	0.0001	***
Tucidinostat 10 µM	3.13	0.27	0.5886	ns

## Data Availability

The original contributions presented in the study are included in the article/Supplementary Materials, further inquiries can be directed to the corresponding author/s.

## Supplementary Materials

The following supporting information can be downloaded at: https://www.mdpi.com/article/10.3390/cells13060517/s1, Figure S1: Descriptive data for the drug panel for the senescence screen. Figure S2: The chemical structures of compounds that decreased senescent cell load. Figure S3: Chemical structures of compounds that decreased senescent cell load. Figure S4: The chemical structures of compounds that increased senescent cell load. Figure S5: Dendrogram indicating structure-function similarity for compounds influencing senescence. Figure S6: Dendrogram indicating structure-function similarity for compounds influencing senescence. Table S1: Information on compound synonyms, targets and pathways used in a screen for *CDKN2A* gene expression. Table S2: Statistics of the percentage of cells stained for biomarkers in female (F) and male (M) dermal fibroblast cells treated with female synthetic hormones at 10 µM or a DMSO-only control. Table S3: Gene expression data in female (F) dermal fibroblast cells treated with female synthetic hormones at 10 µM or a DMSO-only control. Table S4: Gene expression data in male (M) dermal fibroblast cells treated with female synthetic hormones at 10 µM or a DMSO-only control.
