# Supplementary material for: Repurposing Drugs for Senotherapeutic Effect: Potential Senomorphic Effects of Female Synthetic Hormones
Source: Cells. 2024 Mar 15;13(6):517. doi: 10.3390/cells13060517 (PMC10969307; doi:10.3390/cells13060517)

Repurposing drugs for senotherapeutic effect: potential senomorphic effects of female synthetic hormones

Laura R Bramwell[1], Ryan Frankum[1] & Lorna W Harries[1]*

[1]RNA-Mediated Mechanisms of Disease Group, Department of Clinical and Biomedical Sciences (Medical School), Faculty of Health and Life Sciences, University of Exeter, Exeter, UK

*corresponding author

Correspondence address:

Professor Lorna W Harries
Institute of Biomedical and Clinical Sciences
University of Exeter College of Medicine and Health
RILD Building
Royal Devon and Exeter Hospital
Barrack Road
Exeter
Devon
EX2 5DW
United Kingdom

Correspondence email: L.W.Harries@exeter.ac.uk

ORCID numbers: LRB - 0000-0003-4069-3959, RF - 0000-0002-2665-5625, LWH - 0000-0001-7791-8061

X (formerly known as Twitter): @miRNAgirl, @LauraBramwell1, @TeamRNA

Supplementary information

Supplementary Figure Legends S1-S6

Supplementary Fig. S1: Descriptive data for the drug panel for the senescence screen. A. Type of clinical approval, i.e. compounds approved by the European Medical Association (EMA), the US Food and Drug Administration (FDA) or other countries. B. Research areas linked with each compound. C. Frequency of biological pathways that are targeted by the compounds in the screen. D. Compounds in the panel categorised by target/function.

Supplementary Fig. S2: The chemical structures of compounds that decreased senescent cell load. The chemical structures of compounds that decreased senescent cell load as measured by *CDKN2A* (p16) expression are given above.

Supplementary Fig. S3: Chemical structures of compounds that decreased senescent cell load. The chemical structures of compounds that decreased senescent cell load as measured by SAB staining are given above.

Supplementary Fig. S4: The chemical structures of compounds that increased senescent cell load. The chemical structures of compounds that increased senescent cell load as measured by *CDKN2A* (p16) expression are given above.

Supplementary Fig. S5: Dendrogram indicating structure-function similarity for compounds influencing senescence. Dendrogram constructed using the Tanimoto coefficient to show structural similarity of compounds tested that decreased SAB activity.

Supplementary Fig. S6: Dendrogram indicating structure-function similarity for compounds influencing senescence. Dendrogram constructed using the Tanimoto coefficient to show structural similarity of compounds tested that increased *CDKN2A* gene expression.

Supplementary Tables S1-S4

Supplementary Table S1: Information on compound synonyms, targets and pathways used in a screen for *CDKN2A* gene expression. Information provided from the MedChemExpress FDA Approved Drug Library Plus (MedChemTronica, Stockholm).

Product Name	Synonyms	Target	Pathway
5-Aminosalicylic Acid	Mesalamine; 5-ASA;Mesalazine	NF-κB; PAK; PPAR	Cell Cycle/DNA Damage; Cytoskeleton; NF-κB
5-Azacytidine	Ladakamycin; 5-AzaC;Azacitidine	Autophagy; DNA Methyltransferase; Nucleoside Antimetabolite/Analog	Autophagy; Cell Cycle/DNA Damage; Epigenetics
5-Fluorouracil	5-FU	Nucleoside Antimetabolite/Analog	Cell Cycle/DNA Damage
6-Thioguanine	Thioguanine; 2-Amino-6-purinethiol	Autophagy; Deubiquitinase; SARS-CoV	Anti-infection; Autophagy; Cell Cycle/DNA Damage
Abemaciclib (methanesulfonate)	LY2835219 (methanesulfonate)	CDK	Cell Cycle/DNA Damage
Acalabrutinib	ACP-196	Btk	Protein Tyrosine Kinase/RTK
Acamprosate (calcium)	Calcium N-acetylhomotaurinate	GABA Receptor	Membrane Transporter/Ion Channel; Neuronal Signalling
Aceglutamide	α-N-Acetyl-L-glutamine; N2-Acetylglutamine	Autophagy; Endogenous Metabolite	Autophagy; Metabolic Enzyme/Protease
Aclacinomycin A hydrochloride	Aclarubicin hydrochloride	Proteasome; Topoisomerase	Cell Cycle/DNA Damage; Metabolic Enzyme/Protease
Allantoin	5-Ureidohydantoin	Endogenous Metabolite; Imidazoline Receptor	GPCR/G Protein; Metabolic Enzyme/Protease; Neuronal Signalling
Alpelisib	BYL-719	PI3K	PI3K/Akt/mTOR
Altretamine	ENT-50852; RB-1515;WR-95704	DNA Alkylator/Crosslinker	Cell Cycle/DNA Damage

Amlexanox	AA673; Amoxanox; CHX3673	IKK	NF-κB
Amoxapine	CL-67772	Others	Others
Amsacrine	m-AMSA; acridinyl anisidide	Autophagy; Topoisomerase	Autophagy; Cell Cycle/DNA Damage
Anethole (trithione)		Others	Others
Apatinib	YN968D1	Autophagy; c-Kit; RET; Src; VEGFR	Autophagy; Protein Tyrosine Kinase/RTK
Artemisinin	Qinghaosu; NSC 369397	HCV; Parasite	Anti-infection
Artesunate		STAT	JAK/STAT Signalling; Stem Cell/Wnt
Aspirin	ASA; Acetylsalicylic Acid	Autophagy; COX; Mitophagy	Autophagy; Immunology/Inflammation
Atorvastatin (hemicalcium salt)	CI-981; Atorvastatin hemicalcium	Autophagy; HMG-CoA Reductase (HMGCR)	Autophagy; Metabolic Enzyme/Protease
Balsalazide		Interleukin Related; STAT	Immunology/Inflammation; JAK/STAT Signalling; Stem Cell/Wnt
Baricitinib (phosphate)	INCB028050 (phosphate); LY3009104 (phosphate)	JAK	Epigenetics; JAK/STAT Signalling; Stem Cell/Wnt
Belinostat	PXD101; PX105684	Autophagy; HDAC	Autophagy; Cell Cycle/DNA Damage; Epigenetics
Belotecan (hydrochloride)	CKD-602	Topoisomerase	Cell Cycle/DNA Damage
Bendamustine (hydrochloride)	SDX-105; EP-3101	DNA Alkylator/Crosslinker	Cell Cycle/DNA Damage
Bendazol		NO Synthase	Immunology/Inflammation
Berberine (chloride hydrate)	Natural Yellow 18 (chloride hydrate)	Autophagy; Bacterial; ROS; Topoisomerase	Anti-infection; Autophagy; Cell Cycle/DNA Damage; Protein Tyrosine Kinase/RTK
Bexarotene	LGD1069	Autophagy; RAR/RXR	Autophagy; Metabolic Enzyme/Protease
Bezafibrate	BM15075	PPAR	Cell Cycle/DNA Damage
Binimetinib	MEK162; ARRY-162; ARRY-438162	Autophagy; MEK	Autophagy; MAPK/ERK Pathway

Bleomycin (sulfate)		Bacterial; DNA/RNA Synthesis	Anti-infection; Cell Cycle/DNA Damage
Bortezomib	PS-341; Brotezamide; DPBA; LDP 341; MG 341; Radiciol; NSC 681239	Apoptosis; Autophagy; Proteasome	Apoptosis; Autophagy; Metabolic Enzyme/Protease
Bosutinib	SKI-606	Autophagy; Bcr-Abl; Src	Autophagy; Protein Tyrosine Kinase/RTK
Bremelanotide (Acetate)	PT-141 Acetate	Melanocortin Receptor	GPCR/G Protein; Neuronal Signalling
Bromhexine (hydrochloride)		Autophagy; Reactive Oxygen Species	Autophagy; Immunology/Inflammation; Metabolic Enzyme/Protease; NF-κB
Bucladesine (sodium salt)	Dibutyryl-cAMP sodium salt; DC2797; Sodium dibutyryl cAMP	PKA	Protein Tyrosine Kinase/RTK; Stem Cell/Wnt
Bufexamac	Bufexamic acid	HDAC	Cell Cycle/DNA Damage; Epigenetics
Busulfan		DNA Alkylator/Crosslinker	Cell Cycle/DNA Damage
Cabazitaxel	XRP6258; RPR-116258A; taxoid XRP6258	Autophagy; Microtubule/Tubulin	Autophagy; Cell Cycle/DNA Damage; Cytoskeleton
Cabozantinib	XL184; BMS-907351	c-Kit; c-Met/HGFR; FLT3; TAM Receptor; VEGFR	Protein Tyrosine Kinase/RTK
Caffeic acid		5-Lipoxygenase; Endogenous Metabolite; TRP Channel	Membrane Transporter/Ion Channel; Metabolic Enzyme/Protease; Neuronal Signalling
Capecitabine		DNA/RNA Synthesis; Nucleoside Antimetabolite/Analog	Cell Cycle/DNA Damage
Carbamazepine	CBZ; NSC 169864	Autophagy; Mitophagy; Sodium Channel	Autophagy; Membrane Transporter/Ion Channel
Carmofur	HCFU	Nucleoside Antimetabolite/Analog	Cell Cycle/DNA Damage
Carmustine		DNA Alkylator/Crosslinker	Cell Cycle/DNA Damage

Carprofen		Autophagy; COX; FAAH	Autophagy; Immunology/Inflammation; Metabolic Enzyme/Protease; Neuronal Signalling
Ceritinib	LDK378	ALK; IGF-1R; Insulin Receptor	Protein Tyrosine Kinase/RTK
Chlorambucil	CB-1348; WR-139013	DNA Alkylator/Crosslinker	Cell Cycle/DNA Damage
Chloroquine (diphosphate)		Autophagy; Toll-like Receptor (TLR)	Autophagy; Immunology/Inflammation
Chlorpheniramine (maleate)	Chlorphenamine maleate	Histamine Receptor	GPCR/G Protein; Immunology/Inflammation; Neuronal Signalling
Chlorzoxazone		Cytochrome P450	Metabolic Enzyme/Protease
Choline Fenofibrate	ABT-335	Cytochrome P450; PPAR	Cell Cycle/DNA Damage; Metabolic Enzyme/Protease
Chromocarb	Chromone-2-carboxylic acid	Others	Others
Ciprofibrate	Win35833	PPAR	Cell Cycle/DNA Damage
Citalopram (hydrobromide)	(±)-Citalopram hydrobromide; Lu 10-171	Autophagy; Serotonin Transporter	Autophagy; Neuronal Signalling
Citric acid		Apoptosis; Endogenous Metabolite	Apoptosis; Metabolic Enzyme/Protease
Clioquinol	Iodochlorhydroxyquin	Autophagy; Fungal; Mitophagy	Anti-infection; Autophagy
Clofarabine		Autophagy; Nucleoside Antimetabolite/Analog	Autophagy; Cell Cycle/DNA Damage
Clofibrate		PPAR	Cell Cycle/DNA Damage
Cobimetinib	GDC-0973; XL518	MEK	MAPK/ERK Pathway
Colchicine		Autophagy; Microtubule/Tubulin	Autophagy; Cell Cycle/DNA Damage; Cytoskeleton
Conivaptan (hydrochloride)	YM 087	Vasopressin Receptor	GPCR/G Protein

Crizotinib (hydrochloride)	PF-02341066 hydrochloride	ALK; Autophagy; c-Met/HGFR	Autophagy; Protein Tyrosine Kinase/RTK
Cytarabine	Cytosine β-D-arabinofuranoside; Cytosine Arabinoside; Ara-C	Autophagy; DNA/RNA Synthesis; Nucleoside Antimetabolite/Analog	Autophagy; Cell Cycle/DNA Damage
Dabrafenib	GSK2118436A; GSK2118436	Raf	MAPK/ERK Pathway
Dacarbazine	Imidazole Carboxamide	Nucleoside Antimetabolite/Analog	Cell Cycle/DNA Damage
Dacomitinib	PF-00299804; PF-299804	EGFR	JAK/STAT Signalling; Protein Tyrosine Kinase/RTK
Dasatinib (hydrochloride)	BMS 354825 hydrochloride	Autophagy; Bcr-Abl; Src	Autophagy; Protein Tyrosine Kinase/RTK
Daunorubicin (Hydrochloride)	RP 13057 (Hydrochloride); Daunomycin (Hydrochloride); Rubidomycin (Hydrochloride)	ADC Cytotoxin; Autophagy; DNA/RNA Synthesis; Topoisomerase	Antibody-drug Conjugate/ADC Related; Autophagy; Cell Cycle/DNA Damage
Decitabine	NSC 127716; 5-Aza-2'-deoxycytidine	DNA Methyltransferase	Epigenetics
Deferoxamine (mesylate)	Desferrioxamine B mesylate; DFOM	Amyloid-β; Autophagy; Mitophagy	Autophagy; Neuronal Signalling
Dexamethasone	Hexadecadrol; Prednisolone F	Autophagy; Glucocorticoid Receptor; Mitophagy	Autophagy; GPCR/G Protein
Diacerein	Diacerhein; Diacetylrhein	Interleukin Related	Immunology/Inflammation
Diethylstilboestrol	DES; Diethylstilbestrol; Stilbestrol	Estrogen Receptor/ERR	Others
Dimethyl fumarate	DMF	Keap1-Nrf2	NF-κB

Disulfiram	Tetraethylthiuram disulfide; TETD	Aldehyde Dehydrogenase (ALDH); Apoptosis; Interleukin Related	Apoptosis; Immunology/Inflammation; Metabolic Enzyme/Protease
Docetaxel	RP-56976	Microtubule/Tubulin	Cell Cycle/DNA Damage; Cytoskeleton
Doxifluridine	Ro 21-9738; 5-Fluoro-5'-deoxyuridine; 5'-DFUR	Nucleoside Antimetabolite/Analog	Cell Cycle/DNA Damage
Doxorubicin (hydrochloride)	Hydroxydaunorubicin (hydrochloride)	ADC Cytotoxin; AMPK; Autophagy; Mitophagy; Topoisomerase	Antibody-drug Conjugate/ADC Related; Autophagy; Cell Cycle/DNA Damage; Epigenetics; PI3K/Akt/mTOR
Dronedarone	SR 33589	Autophagy; mAChR	Autophagy; GPCR/G Protein; Neuronal Signalling
Duvelisib	IPI-145; INK1197	PI3K	PI3K/Akt/mTOR
Emtricitabine	BW1592	HIV; Reverse Transcriptase	Anti-infection
Enasidenib	AG-221	Isocitrate Dehydrogenase (IDH)	Metabolic Enzyme/Protease
Entacapone		COMT	Metabolic Enzyme/Protease; Neuronal Signalling
Entrectinib	NMS-E628; RXDX-101	ALK; Autophagy; ROS; Trk Receptor	Autophagy; Neuronal Signalling; Protein Tyrosine Kinase/RTK
Epirubicin (hydrochloride)	4'-Epidoxorubicin hydrochloride	Topoisomerase	Cell Cycle/DNA Damage
Erdosteine	RV 144	NF-κB	NF-κB
Erlotinib	CP-358774; NSC 718781; OSI-774	Autophagy; EGFR	Autophagy; JAK/STAT Signalling; Protein Tyrosine Kinase/RTK
Erlotinib (Hydrochloride)	CP-358774 (Hydrochloride); NSC 718781 (Hydrochloride); OSI-774 (Hydrochloride)	Autophagy; EGFR	Autophagy; JAK/STAT Signalling; Protein Tyrosine Kinase/RTK
Estramustine (phosphate sodium)		Microtubule/Tubulin	Cell Cycle/DNA Damage; Cytoskeleton
Ethamsylate		Prostaglandin Receptor	GPCR/G Protein

Ethynyl Estradiol	17α-Ethynylestradiol; Ethynylestradiol	Endogenous Metabolite; Estrogen Receptor/ERR	Metabolic Enzyme/Protease; Others
Etoposide	VP-16; VP-16-213	Apoptosis; Autophagy; Mitophagy; Topoisomerase	Apoptosis; Autophagy; Cell Cycle/DNA Damage
Ezetimibe	SCH 58235	Autophagy; Keap1-Nrf2	Autophagy; NF-κB
Fasudil (Hydrochloride)	HA-1077 (Hydrochloride); AT-877 (Hydrochloride)	Autophagy; Calcium Channel; ROCK	Autophagy; Cell Cycle/DNA Damage; Membrane Transporter/Ion Channel; Neuronal Signalling; Stem Cell/Wnt; TGF-beta/Smad
Favipiravir	T-705	DNA/RNA Synthesis	Cell Cycle/DNA Damage
Fenofibrate		Autophagy; Cytochrome P450; PPAR	Autophagy; Cell Cycle/DNA Damage; Metabolic Enzyme/Protease
Fenofibric acid	FNF acid	COX; PPAR	Cell Cycle/DNA Damage; Immunology/Inflammation
Fingolimod	FTY720 free base	LPL Receptor; PAK	Cell Cycle/DNA Damage; Cytoskeleton; GPCR/G Protein
Floxuridine	5-Fluorouracil 2'-deoxyriboside	Nucleoside Antimetabolite/Analog	Cell Cycle/DNA Damage
Flubendazole		Apoptosis; Microtubule/Tubulin; Parasite	Anti-infection; Apoptosis; Cell Cycle/DNA Damage; Cytoskeleton
Fludarabine	F-ara-A; NSC 118218	DNA/RNA Synthesis; Nucleoside Antimetabolite/Analog; STAT	Cell Cycle/DNA Damage; JAK/STAT Signalling; Stem Cell/Wnt
Fluvastatin (sodium)	XU 62320 sodium	Autophagy; HMG-CoA Reductase (HMGCR)	Autophagy; Metabolic Enzyme/Protease
Folic acid	Vitamin B9; Vitamin M	DNA/RNA Synthesis; Endogenous Metabolite	Cell Cycle/DNA Damage; Metabolic Enzyme/Protease
Forodesine (hydrochloride)	BCX-1777; Immucillin-H hydrochloride	Nucleoside Antimetabolite/Analog	Cell Cycle/DNA Damage
Fostamatinib Disodium	R788(Disodium)	Syk	Protein Tyrosine Kinase/RTK

Gefitinib (hydrochloride)	ZD-1839 hydrochloride	EGFR	JAK/STAT Signalling; Protein Tyrosine Kinase/RTK
Gemcitabine	NSC 613327; LY188011	Autophagy; DNA/RNA Synthesis; Nucleoside Antimetabolite/Analog	Autophagy; Cell Cycle/DNA Damage
Gemfibrozil	CI-719	Cytochrome P450; PPAR	Cell Cycle/DNA Damage; Metabolic Enzyme/Protease
Glasdegib	PF-04449913	Smo	Stem Cell/Wnt
Glycerol phenylbutyrate	HPN-100	Others	Others
Homoharringtonine	Omacetaxine mepesuccinate; HHT	STAT	JAK/STAT Signalling; Stem Cell/Wnt
Hydrocortisone	Cortisol	Endogenous Metabolite; Glucocorticoid Receptor	GPCR/G Protein; Metabolic Enzyme/Protease
Hydroxychloroquine sulfate	HCQ sulfate	Autophagy; Parasite; Toll-like Receptor (TLR)	Anti-infection; Autophagy; Immunology/Inflammation
Hydroxyfasudil	HA-1100	ROCK	Cell Cycle/DNA Damage; Stem Cell/Wnt; TGF-beta/Smad
Hydroxyurea	Hydroxycarbamide	Apoptosis; Autophagy; DNA/RNA Synthesis	Apoptosis; Autophagy; Cell Cycle/DNA Damage
Ibuprofen	(±)-Ibuprofen	COX	Immunology/Inflammation
Idarubicin (hydrochloride)	4-Demethoxydaunorubicin hydrochloride	Autophagy; Topoisomerase	Autophagy; Cell Cycle/DNA Damage
Idelalisib	CAL-101; GS-1101	Autophagy; PI3K	Autophagy; PI3K/Akt/mTOR
Imatinib	STI571; CGP-57148B	Autophagy; Bcr-Abl; c-Kit; PDGFR	Autophagy; Protein Tyrosine Kinase/RTK
Indomethacin	Indometacin	Autophagy; COX	Autophagy; Immunology/Inflammation
Irinotecan (hydrochloride)	CPT-11 hydrochloride; Camptothecin 11 hydrochloride	Autophagy; Topoisomerase	Autophagy; Cell Cycle/DNA Damage

Isotretinoin	13-cis-Retinoic acid	Autophagy; Endogenous Metabolite; RAR/RXR	Autophagy; Metabolic Enzyme/Protease
Ixabepilone	Azaepothilone B; BMS 247550;BMS 247550-1	Apoptosis; Microtubule/Tubulin	Apoptosis; Cell Cycle/DNA Damage; Cytoskeleton
Lamivudine	BCH-189	HIV; Reverse Transcriptase	Anti-infection
Lanatoside C		Autophagy	Autophagy
Lapatinib	GW572016	Autophagy; EGFR	Autophagy; JAK/STAT Signalling; Protein Tyrosine Kinase/RTK
LCZ696	Sacubitril mixture with Valsartan	Angiotensin Receptor; JNK; Neprilysin; NF-κB; p38 MAPK	GPCR/G Protein; MAPK/ERK Pathway; Metabolic Enzyme/Protease; NF-κB
L-Epinephrine (Bitartrate)	(-)-Epinephrine (+)-bitartrate salt; L-Adrenaline (+)-bitartrate salt	Adrenergic Receptor	GPCR/G Protein; Neuronal Signalling
Levoleucovorin (Calcium)	Calcium levofolinate; CL307782	Antifolate	Cell Cycle/DNA Damage
Lidocaine (hydrochloride)	Lignocaine hydrochloride	ERK; MEK; NF-κB; Sodium Channel	MAPK/ERK Pathway; Membrane Transporter/Ion Channel; NF-κB; Stem Cell/Wnt
Loperamide (hydrochloride)	R-18553 (hydrochloride)	Autophagy; Opioid Receptor	Autophagy; GPCR/G Protein; Neuronal Signalling
Lovastatin	Mevinolin	Autophagy; HMG-CoA Reductase (HMGCR)	Autophagy; Metabolic Enzyme/Protease
Melphalan	L-PAM	DNA Alkylator/Crosslinker	Cell Cycle/DNA Damage
Memantine (hydrochloride)	D-145 (hydrochloride)	Autophagy; Cytochrome P450; iGluR	Autophagy; Membrane Transporter/Ion Channel; Metabolic Enzyme/Protease; Neuronal Signalling
Metformin (hydrochloride)	1,1-Dimethylbiguanide hydrochloride	AMPK; Autophagy; Mitophagy	Autophagy; Epigenetics; PI3K/Akt/mTOR

Methotrexate	Amethopterin; CL14377;WR19039	ADC Cytotoxin; Antifolate	Antibody-drug Conjugate/ADC Related; Cell Cycle/DNA Damage
Methylthiouracil	MTU	ERK; Interleukin Related; NF-κB; TNF Receptor	Apoptosis; Immunology/Inflammation; MAPK/ERK Pathway; NF-κB; Stem Cell/Wnt
Metyrapone	Su-4885	Autophagy; Cytochrome P450	Autophagy; Metabolic Enzyme/Protease
Mifepristone	RU486; RU 38486	Autophagy; Glucocorticoid Receptor; Progesterone Receptor	Autophagy; GPCR/G Protein; Others
Miltefosine	HePC; Hexadecyl phosphocholine	Akt; HIV	Anti-infection; PI3K/Akt/mTOR
Mitomycin C	Ametycine	ADC Cytotoxin; Autophagy; DNA Alkylator/Crosslinker; DNA/RNA Synthesis	Antibody-drug Conjugate/ADC Related; Autophagy; Cell Cycle/DNA Damage
Mitoxantrone	Mitozantrone	PKC; Topoisomerase	Cell Cycle/DNA Damage; Epigenetics; TGF-beta/Smad
Mizoribine	NSC 289637; HE 69	HSP	Cell Cycle/DNA Damage; Metabolic Enzyme/Protease
Montelukast (sodium)	MK0476	Autophagy; Leukotriene Receptor	Autophagy; GPCR/G Protein
Mycophenolic acid	Mycophenolate	Others	Others
Nedaplatin	NSC 375101D	DNA/RNA Synthesis	Cell Cycle/DNA Damage
Nefopam (hydrochloride)	Fenazoxine hydrochloride	β-catenin	Stem Cell/Wnt
Nelarabine	506U78; GW 506U78; Nelzarabine	Nucleoside Antimetabolite/Analog	Cell Cycle/DNA Damage
Neratinib	HKI-272	EGFR	JAK/STAT Signalling; Protein Tyrosine Kinase/RTK
Niclosamide	BAY2353	STAT	JAK/STAT Signalling; Stem Cell/Wnt

Nicotinamide	Niacinamide; Nicotinic acid amide; Vitamin B3	Endogenous Metabolite; Sirtuin	Cell Cycle/DNA Damage; Epigenetics; Metabolic Enzyme/Protease
Nifuroxazide		STAT	JAK/STAT Signalling; Stem Cell/Wnt
Niraparib	MK-4827	PARP	Cell Cycle/DNA Damage; Epigenetics
Nitisinone	NTBC; Nitisone;SC0735	Reactive Oxygen Species	Immunology/Inflammation; Metabolic Enzyme/Protease; NF-κB
Olaparib	AZD2281; KU0059436	Autophagy; Mitophagy; PARP	Autophagy; Cell Cycle/DNA Damage; Epigenetics
Opicapone	BIA 9-1067	COMT	Metabolic Enzyme/Protease; Neuronal Signalling
Orotic acid	6-Carboxyuracil; Vitamin B13	Endogenous Metabolite; Nucleoside Antimetabolite/Analog	Cell Cycle/DNA Damage; Metabolic Enzyme/Protease
Osalmid	Oxaphenamide; 4'-Hydroxysalicylanilide	HBV	Anti-infection
Paclitaxel	Taxol	ADC Cytotoxin; Autophagy; Microtubule/Tubulin	Antibody-drug Conjugate/ADC Related; Autophagy; Cell Cycle/DNA Damage; Cytoskeleton
Palbociclib (hydrochloride)	PD 0332991 hydrochloride	CDK	Cell Cycle/DNA Damage
Pamidronic acid		Wnt; β-catenin	Stem Cell/Wnt
Panobinostat	LBH589; NVP-LBH589	Autophagy; HDAC	Autophagy; Cell Cycle/DNA Damage; Epigenetics
Pazopanib	GW786034	Autophagy; c-Kit; FGFR; PDGFR; VEGFR	Autophagy; Protein Tyrosine Kinase/RTK
Pazopanib (Hydrochloride)	GW786034 (Hydrochloride)	Autophagy; c-Fms; c-Kit; FGFR; PDGFR; VEGFR	Autophagy; Protein Tyrosine Kinase/RTK
Peficitinib	ASP015K; JNJ-54781532	JAK	Epigenetics; JAK/STAT Signalling; Stem Cell/Wnt
Pemetrexed (disodium hemipenta hydrate)	LY231514 (disodium hemipenta hydrate)	Antifolate; Autophagy	Autophagy; Cell Cycle/DNA Damage

Penfluridol	R-16341	Autophagy; Calcium Channel	Autophagy; Membrane Transporter/Ion Channel; Neuronal Signalling
Pexidartinib	PLX-3397	c-Fms; c-Kit	Protein Tyrosine Kinase/RTK
Phenindione	Rectadione	Others	Others
Pioglitazone	U 72107	PPAR	Cell Cycle/DNA Damage
Pipobroman		Others	Others
Pirfenidone	AMR69	TGF-beta/Smad	Stem Cell/Wnt; TGF-beta/Smad
Pixantrone (dimaleate)	BBR 2778 dimaleate	Topoisomerase	Cell Cycle/DNA Damage
Podofilox	Podophyllotoxin	Microtubule/Tubulin	Cell Cycle/DNA Damage; Cytoskeleton
Ponatinib	AP24534	Autophagy; Bcr-Abl; FGFR; PDGFR; Src; VEGFR	Autophagy; Protein Tyrosine Kinase/RTK
Pralatrexate		Antifolate	Cell Cycle/DNA Damage
Pranlukast	ONO-1078	Leukotriene Receptor	GPCR/G Protein
Procarbazine (Hydrochloride)		DNA Alkylator/Crosslinker	Cell Cycle/DNA Damage
Propranolol (hydrochloride)		Adrenergic Receptor; Autophagy	Autophagy; GPCR/G Protein; Neuronal Signalling
Pyrimethamine	Pirimecidan; Pirimetamin; RP 4753	Antifolate; Parasite	Anti-infection; Cell Cycle/DNA Damage
Pyrvinium pamoate	Pyrvinium embonate	Wnt	Stem Cell/Wnt
Quinapril (hydrochloride)	CI-906	Angiotensin-converting Enzyme (ACE)	Metabolic Enzyme/Protease
Raloxifene (hydrochloride)	LY156758 hydrochloride; LY139481 hydrochloride	Autophagy; Estrogen Receptor/ERR	Autophagy; Others
Raltitrexed	ZD1694; D1694; ICI-D1694	Nucleoside Antimetabolite/Analog; Thymidylate Synthase	Apoptosis; Cell Cycle/DNA Damage
Rasagiline (mesylate)	AGN1135 (mesylate); TVP1012 (mesylate)	Autophagy; Monoamine Oxidase	Autophagy; Neuronal Signalling
Regorafenib (monohydrate)	BAY 73-4506 monohydrate	Autophagy; PDGFR; Raf; RET; VEGFR	Autophagy; MAPK/ERK Pathway; Protein Tyrosine Kinase/RTK

Resveratrol	SRT 501; trans-Resveratrol	Autophagy; IKK; Mitophagy; Sirtuin	Autophagy; Cell Cycle/DNA Damage; Epigenetics; NF-κB
Retinoic acid	ATRA; Tretinoin; Vitamin A acid; all-trans-Retinoic acid	Autophagy; Endogenous Metabolite; PPAR; RAR/RXR	Autophagy; Cell Cycle/DNA Damage; Metabolic Enzyme/Protease
Ribociclib	LEE011	CDK	Cell Cycle/DNA Damage
Ripasudil	K-115	ROCK	Cell Cycle/DNA Damage; Stem Cell/Wnt; TGF-beta/Smad
Rosiglitazone	BRL 49653	Autophagy; PPAR; TRP Channel	Autophagy; Cell Cycle/DNA Damage; Membrane Transporter/Ion Channel; Neuronal Signalling
Rucaparib (phosphate)	AG-014699 phosphate; PF-01367338 phosphate	PARP	Cell Cycle/DNA Damage; Epigenetics
Ruxolitinib	INCB018424	Autophagy; JAK; Mitophagy	Autophagy; Epigenetics; JAK/STAT Signalling; Stem Cell/Wnt
Salicylic acid	2-Hydroxybenzoic acid	Autophagy; COX; Endogenous Metabolite; Mitophagy	Autophagy; Immunology/Inflammation; Metabolic Enzyme/Protease
Selumetinib	AZD6244; ARRY-142886	MEK	MAPK/ERK Pathway
Sertraline (hydrochloride)		Serotonin Transporter	Neuronal Signalling
Sildenafil	UK-92480	Autophagy; Phosphodiesterase (PDE)	Autophagy; Metabolic Enzyme/Protease
Silibinin	Silybin; Silibinin A; Silymarin I	Autophagy; Reactive Oxygen Species	Autophagy; Immunology/Inflammation; Metabolic Enzyme/Protease; NF-κB
Sitagliptin	MK0431	Autophagy; Dipeptidyl Peptidase	Autophagy; Metabolic Enzyme/Protease
Sodium phenylbutyrate	Sodium 4-phenylbutyrate; TriButyrate	Autophagy; HDAC	Autophagy; Cell Cycle/DNA Damage; Epigenetics
Sorafenib	Bay 43-9006	Autophagy; FLT3; Raf; VEGFR	Autophagy; MAPK/ERK Pathway; Protein Tyrosine Kinase/RTK

Sulfasalazine	NSC 667219	Autophagy; NF-κB	Autophagy; NF-κB
Sunitinib	SU 11248	Autophagy; Mitophagy; PDGFR; VEGFR	Autophagy; Protein Tyrosine Kinase/RTK
Tacrolimus (monohydrate)	FK506 (monohydrate); Fujimycin (monohydrate); FR900506 (monohydrate)	Autophagy; FKBP; Phosphatase	Apoptosis; Autophagy; Immunology/Inflammation; Metabolic Enzyme/Protease
Talazoparib	BMN-673; LT-673	PARP	Cell Cycle/DNA Damage; Epigenetics
Tamoxifen	ICI47699; Z-Tamoxifen; trans-Tamoxifen	Autophagy; Oestrogen Receptor/ERR	Autophagy; Others
Taurodeoxychloic Acid (sodium hydrate)	Sodium taurodeoxycholate monohydrate	Apoptosis; Caspase	Apoptosis
Tegafur	FT 207; NSC 148958	Nucleoside Antimetabolite/Analog	Cell Cycle/DNA Damage
Telmisartan	BIBR 277	Angiotensin Receptor; Autophagy	Autophagy; GPCR/G Protein
Temozolomide	NSC 362856; CCRG 81045; TMZ	Autophagy; DNA Alkylator/Crosslinker	Autophagy; Cell Cycle/DNA Damage
Temsirolimus	CCI-779	Autophagy; mTOR	Autophagy; PI3K/Akt/mTOR
Teniposide	VM26	Topoisomerase	Cell Cycle/DNA Damage
Teprenone	Geranylgeranylacetone	HSP	Cell Cycle/DNA Damage; Metabolic Enzyme/Protease
Terazosin (hydrochloride dihydrate)		Adrenergic Receptor	GPCR/G Protein; Neuronal Signalling
Theophylline	1,3-Dimethylxanthine; Theo-24	Adenosine Receptor; Autophagy; Endogenous Metabolite; Phosphodiesterase (PDE)	Autophagy; GPCR/G Protein; Metabolic Enzyme/Protease
Thio-TEPA		DNA Alkylator/Crosslinker	Cell Cycle/DNA Damage

Tofacitinib (citrate)	Tasocitinib citrate; CP-690550 citrate	JAK	Epigenetics; JAK/STAT Signalling; Stem Cell/Wnt
Topotecan (Hydrochloride)	SKF 104864A (Hydrochloride); NSC 609669 (Hydrochloride)	Autophagy; Topoisomerase	Autophagy; Cell Cycle/DNA Damage
Trametinib (DMSO solvate)	GSK-1120212 (DMSO solvate); JTP-74057 (DMSO solvate)	MEK	MAPK/ERK Pathway
Tranylcypromine (hemisulfate)	dl-Tranylcypromine hemisulfate; trans-2-Phenylcyclopropylamine hemisulfate salt	Histone Demethylase; Monoamine Oxidase	Epigenetics; Neuronal Signalling
Triclabendazole	CGA89317	Microtubule/Tubulin	Cell Cycle/DNA Damage; Cytoskeleton
Trifluridine	Trifluorothymidine; 5-Trifluorothymidine; TFT	HSV; Nucleoside Antimetabolite/Analog; Thymidylate Synthase	Anti-infection; Apoptosis; Cell Cycle/DNA Damage
Trimethoprim		Antifolate	Cell Cycle/DNA Damage
Troglitazone	CS-045	Autophagy; PPAR	Autophagy; Cell Cycle/DNA Damage
Troxipide		Others	Others
Tucidinostat	Chidamide; HBI-8000;CS 055	HDAC	Cell Cycle/DNA Damage; Epigenetics
Upadacitinib	ABT-494	JAK	Epigenetics; JAK/STAT Signalling; Stem Cell/Wnt
VAL-083	Dianhydrodulcitol; Dianhydrogalactitol	DNA Alkylator/Crosslinker	Cell Cycle/DNA Damage
Valproic acid (sodium salt)	Sodium Valproate	Autophagy; HDAC; Mitophagy	Autophagy; Cell Cycle/DNA Damage; Epigenetics
Valpromide		Others	Others
Valrubicin	AD-32	PKC	Epigenetics; TGF-beta/Smad
Vemurafenib	PLX4032; RG7204; RO5185426	Autophagy; Raf	Autophagy; MAPK/ERK Pathway

Venetoclax	ABT-199; GDC-0199	Autophagy; Bcl-2 Family	Apoptosis; Autophagy
Vidarabine	Ara-A; Adenine Arabinoside; 9-β-D-Arabinofuranosyladenine	HSV; Nucleoside Antimetabolite/Analog	Anti-infection; Cell Cycle/DNA Damage
Vinblastine (sulfate)	Vincaleukoblastine sulfate salt	Autophagy; Microtubule/Tubulin	Autophagy; Cell Cycle/DNA Damage; Cytoskeleton
Vinorelbine (ditartrate)	KW-2307; Nor-5'-anhydrovinblastine ditartrate	Autophagy; Microtubule/Tubulin	Autophagy; Cell Cycle/DNA Damage; Cytoskeleton
Vorinostat	SAHA	Autophagy; HDAC; Mitophagy	Autophagy; Cell Cycle/DNA Damage; Epigenetics
Zidovudine	Azidothymidine; AZT; ZDV	CRISPR/Cas9; HIV	Anti-infection; Cell Cycle/DNA Damage

Supplementary Table S2: Statistics of the percentage of cells stained for biomarkers in female (F) and male (M) dermal fibroblast cells treated with female synthetic hormones at 10 μM or a DMSO-only control. Biomarkers for senescence (senescence-associated beta galactosidase (SAB), proliferation (Ki67) and DNA damage (γH2AX and TUNEL)) are assessed. Although some cells stained for it, γH2AX staining was negligible across all experimental groups. The mean ± standard error of the mean (SEM) and p values from one-way ANOVAs with Fisher's post hoc test are reported. Significant p values > 0.05 are emboldened. n = 3 for all groups.

Biomarker	F DSMO		F Diethylstilboestrol			F Ethynyl estradiol			F Levonorgestrel		
	Mean	SEM	Mean	SEM	p value	Mean	SEM	p value	Mean	SEM	p value
SA-B-GAL	18.21	2.7040	19.12	1.2410	0.8485	10.12	1.2400	0.1022	17.68	3.9700	0.9099
Ki67	22.66	3.7140	18.24	3.0860	0.3822	20.54	4.0410	0.6722	27.66	2.1510	0.3245
γH2AX	0.00	0.0000	0.00	0.0000	1.0000	0.00	0.0000	1.0000	0.00	0.0000	1.0000
TUNEL	0.93	0.7201	1.45	1.4500	0.8140	4.50	2.4830	0.1185	2.93	2.2740	0.3682
Biomarker	M DMSO		M Diethylstilboestrol			M Ethynyl estradiol			M Levonorgestrel		
	Mean	SEM	Mean	SEM	p value	Mean	SEM	p value	Mean	SEM	p value
SA-B-GAL	44.17	7.5060	30.98	1.5070	**0.0122**	30.10	1.3170	**0.0083**	21.54	0.8541	**0.0002**
Ki67	26.04	2.7720	14.23	3.5600	**0.0289**	22.80	4.5130	0.5190	26.52	3.4300	0.9235
γH2AX	0.00	0.0000	0.00	0.0000	1.0000	0.00	0.0000	1.0000	0.00	0.0000	1.0000
TUNEL	0.74	0.1738	1.09	0.8579	0.8757	4.30	1.8790	0.1191	1.23	0.6385	0.8246

Supplementary Table S3: Gene expression data in female (F) dermal fibroblast cells treated with female synthetic hormones at 10 µM or a DMSO-only control. Genes relating to apoptosis, senescence, senescence-associated secretory phenotype (SASP) factors, splicing factors and spliceosomal components are assessed. The mean ± standard error of the mean (SEM) and p values from one-way ANOVAs with Fisher's post hoc test are reported. Significant p values > 0.05 are emboldened. n = 3 for all groups.

Gene	F DSMO		F Diethylstilboestrol			F Ethynyl estradiol			F Levonorgestrel		
	Mean	SEM	Mean	SEM	p value	Mean	SEM	p value	Mean	SEM	p value
AKAP17A	0.0000	0.04877	-0.0696	0.06734	0.8157	-0.0861	0.19660	0.7730	-0.6028	0.33060	0.0568
ATM	0.0000	0.09368	0.1328	0.04374	0.5080	0.3512	0.01997	0.0922	0.2723	0.14300	0.1838
BCL2	0.0000	0.29700	-0.1545	0.14940	0.6721	0.8249	0.26470	**0.0351**	0.9137	0.49020	**0.0214**
CASP1	0.0000	0.05322	-0.1025	0.07671	0.5717	0.0087	0.18090	0.9618	-0.0475	0.24950	0.7926
CASP3	0.0000	0.14620	0.0350	0.02214	0.8806	0.2660	0.09735	0.2631	0.2579	0.26520	0.2772
CASP7	0.0000	0.12580	0.2608	0.08696	0.4091	0.4815	0.01044	0.1372	0.2679	0.22760	0.3968
CASP8	0.0000	0.07224	-0.3233	0.06252	**0.0643**	0.1314	0.08837	0.4311	0.0459	0.11180	0.7816
CASP9	0.0000	0.10600	0.0008	0.05213	0.9962	0.0991	0.15160	0.5748	0.1031	0.10360	0.5596
CXCL1	0.0000	0.02680	-0.1083	0.06449	0.3681	0.3101	0.09798	0.0174	0.4028	0.17070	0.0033
CXCL10	0.0000	0.05642	4.7470	4.55000	0.3820	5.5460	5.34900	0.3092	14.1800	0.28910	**0.0162**
HNRNPA0	0.0000	0.13760	-0.0999	0.16080	0.6152	-0.0924	0.04577	0.6418	-0.1178	0.19000	0.5541
HNRNPA1	0.0000	0.05818	-0.1571	0.07813	0.3730	-0.0338	0.15350	0.8461	0.1071	0.03450	0.5409
HNRNPA2B1	0.0000	0.06352	0.1345	0.04062	0.4240	0.2929	0.11460	0.0931	0.2909	0.18700	0.0951
HNRNPD	0.0000	0.07285	0.0286	0.09641	0.8892	0.3005	0.13140	0.1563	0.2270	0.18050	0.2777
HNRNPH3	0.0000	0.08328	-0.0232	0.26490	0.9271	0.1767	0.32310	0.4884	0.4416	0.17970	0.0955
HNRNPK	0.0000	0.06065	-0.7314	0.07480	**0.0003**	0.1734	0.11820	0.2883	0.2710	0.08945	0.1053
HNRNPM	0.0000	0.46980	-0.0369	0.26360	0.9469	-0.1685	0.37730	0.7610	0.3877	0.18980	0.4867
HNRNPUL2	0.0000	0.03668	0.0272	0.03475	0.8521	0.0702	0.11220	0.6310	0.1518	0.10880	0.3057
IFNy	0.0000	0.05642	0.2689	0.10360	0.2016	0.3659	0.17120	0.0887	0.3534	0.17230	0.0992
IL10	0.0000	4.51400	-0.0561	4.20500	0.9857	-4.0910	0.17120	0.2042	-4.1040	0.17230	0.2028
IL12A	0.0000	0.05474	0.1378	0.02569	0.3868	0.2102	0.05250	0.1935	0.4231	0.07220	**0.0148**
IL12B	0.0000	4.64600	4.9630	4.76200	0.4270	4.8630	4.62700	0.4362	9.6640	0.35930	0.1320
IL1B	0.0000	0.21700	0.0328	0.34880	0.9524	0.2431	0.52080	0.6593	0.6907	0.09538	0.2200

IL2	0.0000	0.05642	0.2689	0.10360	0.2016	0.3659	0.17120	0.0887	0.3534	0.17230	0.0992
IL6	0.0000	0.32160	0.1824	0.14410	0.5236	0.8914	0.36240	**0.0057**	0.4181	0.11000	0.1544
IL8	0.0000	0.13850	0.0514	0.04925	0.8299	0.7404	0.17170	**0.0062**	0.4335	0.27220	0.0841
MMP1	0.0000	0.03852	-0.2345	0.13800	0.0792	-0.2480	0.14960	0.0649	0.2607	0.05976	0.0535
MMP3	0.0000	0.12630	0.0219	0.02824	0.9255	0.4238	0.13130	0.0849	0.3824	0.19750	0.1169
MMP9	0.0000	4.71600	0.4995	4.79000	0.9365	-4.2940	0.17120	0.4966	4.9560	4.46200	0.4338
NOVA1	0.0000	0.32020	0.4971	0.03800	0.0645	0.7833	0.10840	**0.0065**	0.7211	0.08223	**0.0109**
PNISR	0.0000	0.09611	0.2793	0.03049	0.1691	0.4409	0.18140	**0.0372**	0.4214	0.09260	**0.0452**
SRSF1	0.0000	0.04145	0.0309	0.08961	0.8674	0.0617	0.16330	0.7388	0.0056	0.14670	0.9759
SRSF2	0.0000	0.19890	0.0541	0.19900	0.8910	-0.2066	0.22320	0.6027	0.0718	0.26780	0.8559
SRSF3	0.0000	0.08635	0.1872	0.06322	0.1571	0.1734	0.07024	0.1880	0.1542	0.09125	0.2389
SRSF6	0.0000	0.04241	0.0692	0.02897	0.5374	0.1745	0.06653	0.1317	-0.0079	0.14760	0.9435
SRSF7	0.0000	0.12380	-0.0898	0.27420	0.6773	0.2905	0.07296	0.1891	0.0239	0.21770	0.9116
TNFα	0.0000	4.49500	-0.3955	4.06500	0.9171	-0.1749	4.30400	0.9633	4.6620	0.75530	0.2304
TNFβ	0.0000	0.49780	-4.6310	4.58600	0.3420	-4.0960	4.53000	0.3992	-8.5940	4.85000	0.0879
TRA2B	0.0000	0.07368	0.0641	0.03576	0.6262	0.0000	0.15040	0.9998	-0.0894	0.13670	0.4985

Supplementary Table S4: Gene expression data in male (M) dermal fibroblast cells treated with female synthetic hormones at 10 µM or a DMSO-only control. Genes relating to apoptosis, senescence, senescence-associated secretory phenotype (SASP) factors, splicing factors and spliceosomal components are assessed. The mean ± standard error of the mean (SEM) and p values from one-way ANOVAs with Fisher's post hoc test are reported. Significant p values > 0.05 are emboldened. n = 3 for all groups.

Gene	M DMSO		M Diethylstilboestrol			M Ethynyl estradiol			M Levonorgestrel		
	Mean	SEM	Mean	SEM	p value	Mean	SEM	p value	Mean	SEM	p value
AKAP17A	0.0000	0.37760	0.0210	0.09971	0.9440	-0.3786	0.04596	0.2155	0.0548	0.18780	0.8543
ATM	0.0000	0.18660	-0.2546	0.25860	0.2124	0.2516	0.11400	0.2176	0.3945	0.08646	0.0613
BCL2	0.0000	0.08764	0.0840	0.05186	0.8177	-0.1902	0.20160	0.6028	0.2631	0.20420	0.4734
CASP1	0.0000	0.05472	-0.2825	0.08580	0.1312	-0.2063	0.09652	0.2625	-0.2590	0.05251	0.1641
CASP3	0.0000	0.15720	0.0125	0.13620	0.9574	0.2428	0.24680	0.3054	0.5133	0.06678	**0.0397**
CASP7	0.0000	0.37070	-0.0541	0.18840	0.8625	-0.3383	0.30450	0.2878	0.1789	0.19410	0.5689
CASP8	0.0000	0.07372	-0.0977	0.17910	0.5567	-0.0275	0.14140	0.8679	0.2294	0.13750	0.1777
CASP9	0.0000	0.09776	-0.0735	0.11170	0.6768	0.0691	0.05622	0.6950	-0.0901	0.21660	0.6099
CXCL1	0.0000	0.04399	0.1552	0.05730	0.2029	0.0829	0.07512	0.4884	0.1873	0.01454	0.1288
CXCL10	0.0000	0.26310	4.2230	4.56800	0.4356	4.7290	4.73100	0.3837	4.5990	4.33400	0.3967
HNRNPA0	0.0000	0.23390	0.2974	0.05454	0.1464	0.2161	0.05255	0.2837	-0.0282	0.09186	0.8867
HNRNPA1	0.0000	0.22000	-0.0813	0.09392	0.6417	0.0273	0.06772	0.8755	0.0037	0.14640	0.9832
HNRNPA2B1	0.0000	0.15400	-0.0063	0.09194	0.9698	0.0778	0.02540	0.6415	0.2511	0.14480	0.1452
HNRNPD	0.0000	0.13050	0.0631	0.08105	0.7590	-0.2701	0.23930	0.1999	0.3851	0.13380	0.0747
HNRNPH3	0.0000	0.07911	-0.0558	0.10670	0.8256	-0.0306	0.08842	0.9037	0.3165	0.09597	0.2223
HNRNPK	0.0000	0.18530	0.5549	0.08881	**0.0029**	0.4991	0.04690	**0.0060**	0.6198	0.15490	**0.0012**
HNRNPM	0.0000	0.66730	0.3579	0.03542	0.5204	0.0038	0.48630	0.9945	0.1567	0.18580	0.7773
HNRNPUL2	0.0000	0.15270	-0.1152	0.07064	0.4334	-0.0466	0.15870	0.7493	-0.0581	0.04227	0.6910
IFNy	0.0000	0.26310	-0.2527	0.09234	0.2286	-0.0330	0.03345	0.8722	0.2254	0.10600	0.2806
IL10	0.0000	0.26310	-0.2527	0.09234	0.9358	-0.0330	0.03345	0.9916	0.2254	0.10600	0.9428
IL12A	0.0000	0.24460	0.0120	0.10710	0.9391	0.0624	0.03350	0.6921	0.2606	0.10890	0.1118
IL12B	0.0000	4.41100	4.3260	4.47200	0.4876	0.2934	4.84600	0.9622	0.1350	4.42100	0.9826

IL1B	0.0000	0.49860	0.2727	0.52560	0.6211	0.4637	0.19580	0.4041	1.0400	0.39830	0.0727
IL2	0.0000	0.26310	-0.2527	0.09234	0.2286	-0.0330	0.03345	0.8722	0.2254	0.10600	0.2806
IL6	0.0000	0.08726	-0.0177	0.05395	0.9503	0.0457	0.08289	0.8723	-0.0176	0.16700	0.9507
IL8	0.0000	0.20190	-0.0505	0.16650	0.8329	-0.1289	0.16130	0.5914	0.1584	0.04279	0.5106
MMP1	0.0000	0.08279	-0.0753	0.08249	0.5556	-0.1072	0.03083	0.4040	-0.0403	0.03862	0.7514
MMP3	0.0000	0.18370	-0.1831	0.14400	0.4390	0.0401	0.07253	0.8643	-0.0985	0.28310	0.6750
MMP9	0.0000	4.82100	4.1040	4.50300	0.5156	0.0306	4.64900	0.9961	0.2664	4.70400	0.9661
NOVA1	0.0000	0.03965	-0.2846	0.33680	0.2722	0.0007	0.07166	0.9977	0.1478	0.08962	0.5632
PNISR	0.0000	0.14170	-0.0921	0.09357	0.6413	-0.0557	0.10550	0.7776	0.2480	0.24270	0.2193
SRSF1	0.0000	0.13730	0.1992	0.07704	0.2899	-0.1585	0.13280	0.3967	0.2535	0.17920	0.1827
SRSF2	0.0000	0.48240	0.2587	0.11900	0.5154	-0.0533	0.13000	0.8928	-0.0508	0.37510	0.8977
SRSF3	0.0000	0.08197	0.0421	0.15530	0.7429	0.1169	0.04936	0.3677	0.1806	0.07489	0.1714
SRSF6	0.0000	0.08067	0.1928	0.03623	0.0983	0.2390	0.05504	**0.0449**	-0.0977	0.09249	0.3868
SRSF7	0.0000	0.09516	-0.2363	0.10150	0.2809	-0.1442	0.07144	0.5057	0.1147	0.10790	0.5956
TNFα	0.0000	0.13420	0.1262	0.17540	0.9735	0.2699	0.08071	0.9434	-0.0307	0.20740	0.9936
TNFβ	0.0000	0.33380	0.4300	0.49070	0.9287	0.5454	0.21720	0.9096	-3.8980	4.87100	0.4219
TRA2B	0.0000	0.04219	0.2672	0.07561	0.0550	0.2555	0.02158	0.0653	0.2954	0.10360	**0.0361**

Supplementary Figures

Supplementary Fig. S1

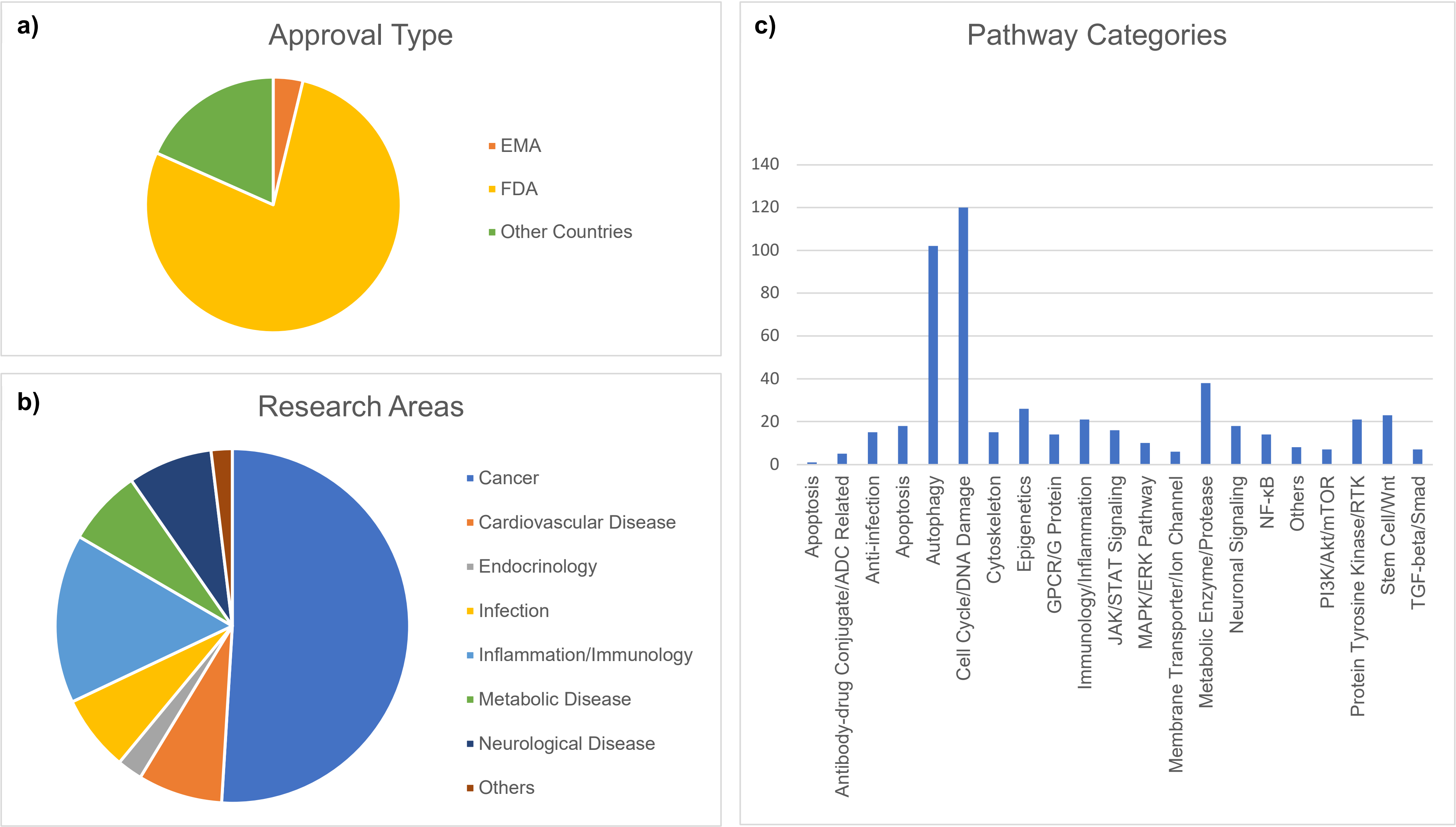

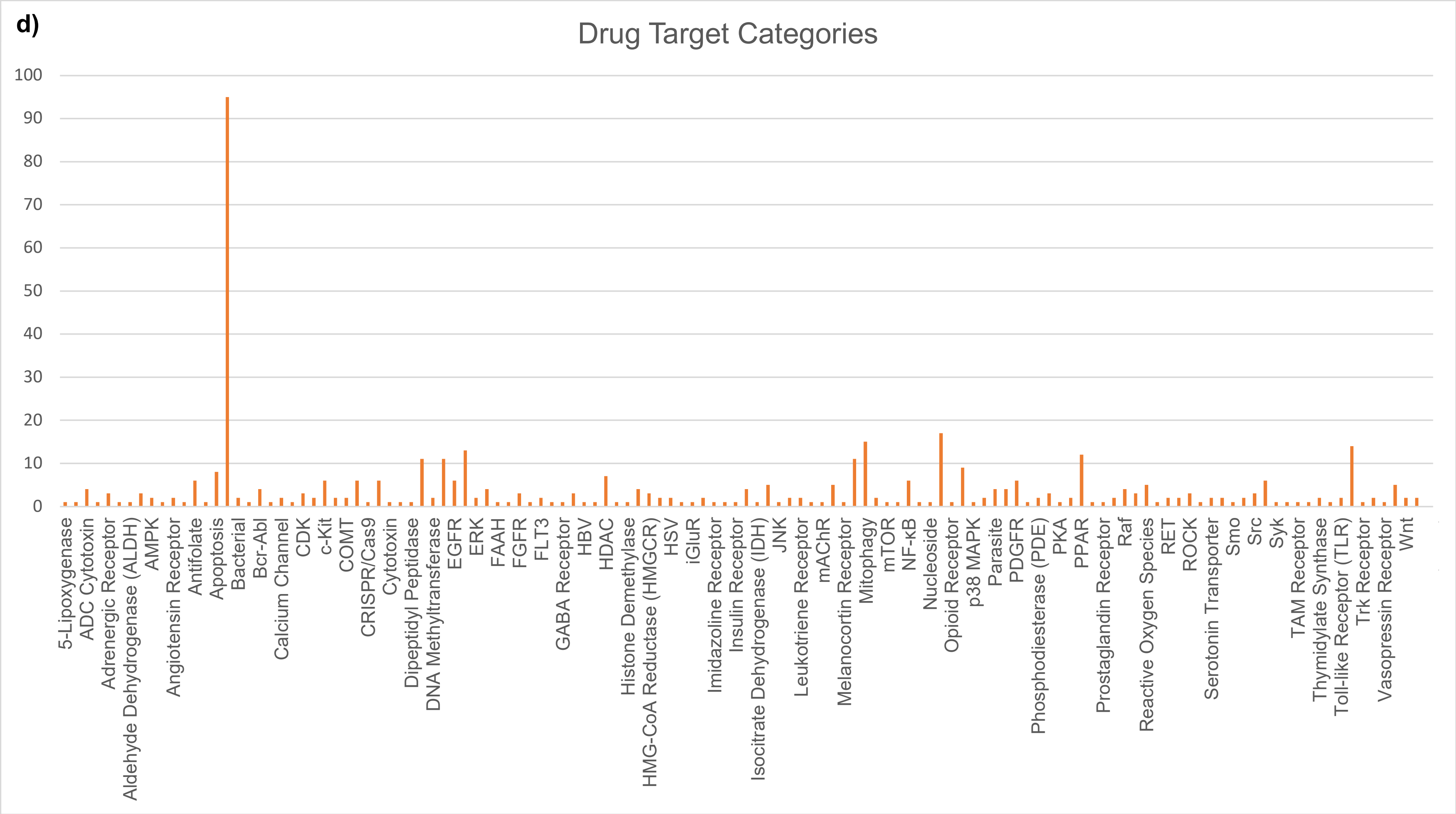
d)
Drug Target Categories
100
90
80
70
60
50
40
30
20
10
0
5-Lipoxygenase
ADC Cytotoxin
Adrenergic Receptor
Aldehyde Dehydrogenase (ALDH)
AMPK
Angiotensin Receptor
Antifolate
Apoptosis
Bacterial
Bcr-Abl
Calcium Channel
CDK
c-Kit
COMT
CRISPR/Cas9
Cytotoxin
Dipeptidyl Peptidase
DNA Methyltransferase
EGFR
ERK
FAAH
FGFR
FLT3
GABA Receptor
HBV
HDAC
Histone Demethylase
HMG-CoA Reductase (HMGCR)
HSV
iGluR
Imidazoline Receptor
Insulin Receptor
Isocitrate Dehydrogenase (IDH)
JNK
Leukotriene Receptor
mAChR
Melanocortin Receptor
Mitophagy
mTOR
NF-κB
Nucleoside
Opioid Receptor
p38 MAPK
Parasite
PDGFR
Phosphodiesterase (PDE)
PKA
PPAR
Prostaglandin Receptor
Raf
Reactive Oxygen Species
RET
ROCK
Serotonin Transporter
Smo
Src
Syk
TAM Receptor
Thymidylate Synthase
Toll-like Receptor (TLR)
Trk Receptor
Vasopressin Receptor
Wnt

Supplementary Fig. S2

5-Azacytidine

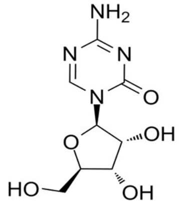

Abemaciclib (methanesulfonate)

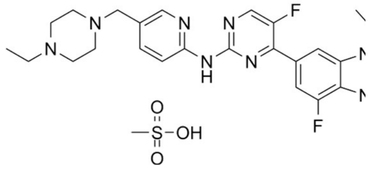

Acalabrutinib

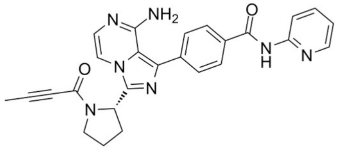

Balsalazide

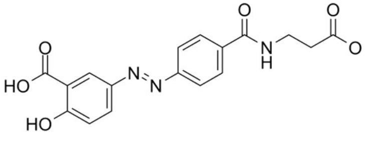

Bendazol

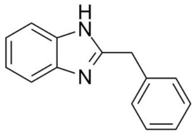

Bexarotene

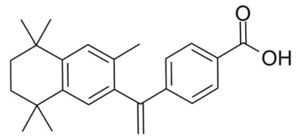

Bortezomib

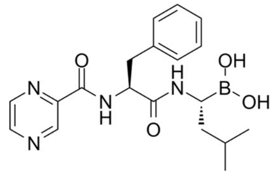

Bosutinib

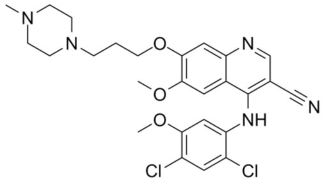

Cabozantinib

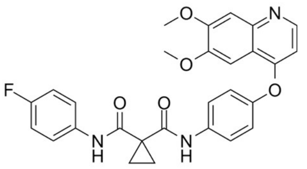

Carmofur

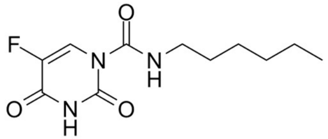

Chlorpheniramine (maleate)

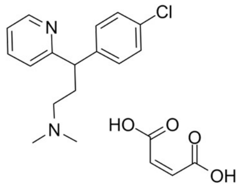

Chlorzoxazone

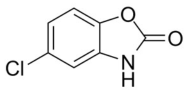

Chromocarb

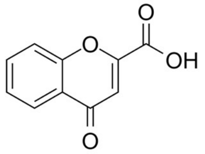

Clofarabine

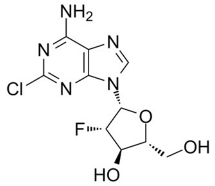

Conivaptan (hydrochloride)

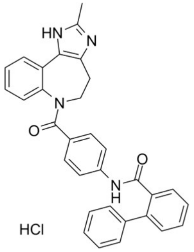

Note this figure continues onto the following page

Erlotinib

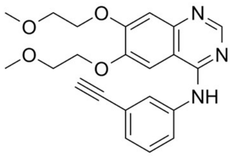

Dexamethasone

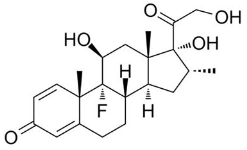

Diethylstilboestrol

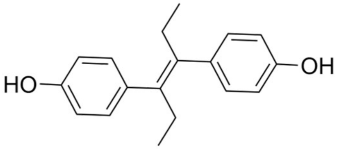

Dimethyl fumarate

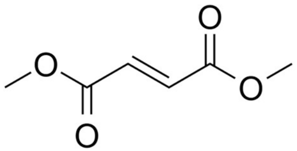

Dronedarone

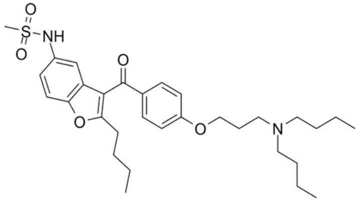

Ethynyl estradiol

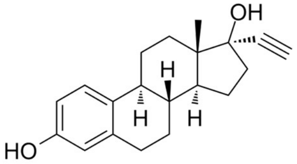

Methylthiouracil

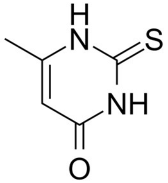

Metyrapone

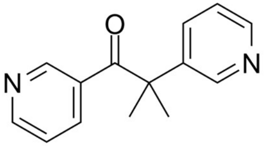

Nefopam (hydrochloride)

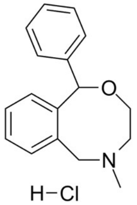

Nelarabine

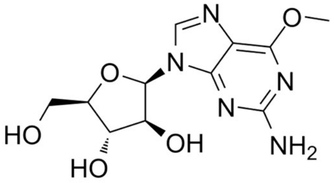

Niraparib

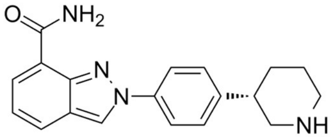

Osalmid

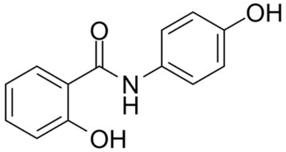

Penfluridol

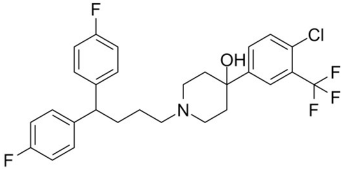

Sodium 4-phenylbutyrate

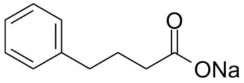

Topotecan (Hydrochloride)

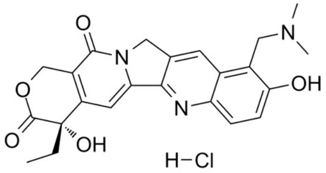

Supplementary Fig. S3

Aspirin

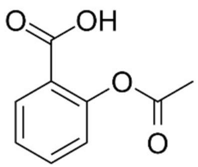

Cabozantinib

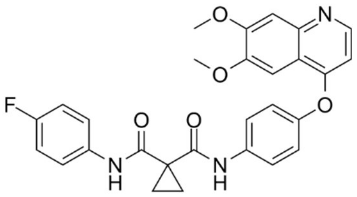

Carmofur

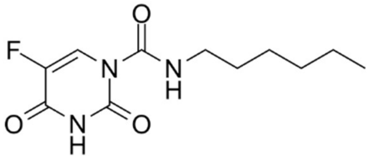

Chlorpheniramine (maleate)

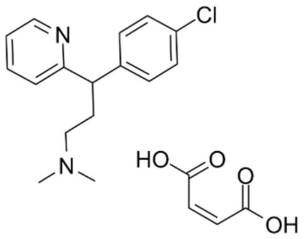

Ethynyl estradiol

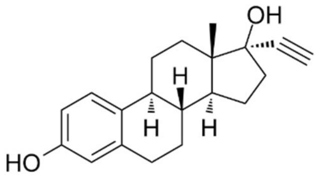

Levonorgestrel

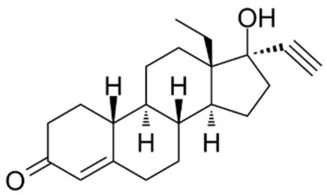

Metyrapone

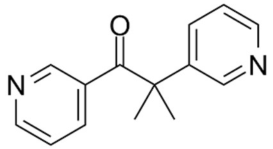

Penfluridol

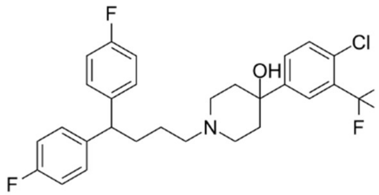

Diethylstilboestrol

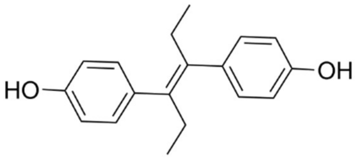

Sodium 4-phenylbutyrate

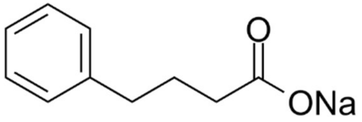

Supplementary Fig. S4

Atorvastatin (hemicalcium salt)

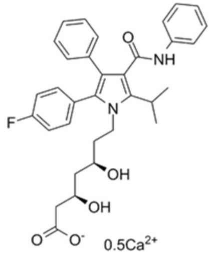

Chlorambucil

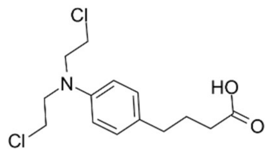

Cobimetinib

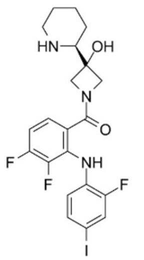

Doxorubicin (hydrochloride)

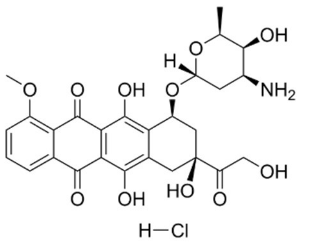

Gemfibrozil

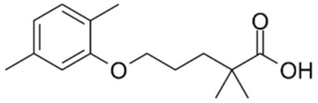

Homoharringtonine

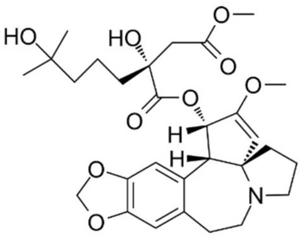

Imatinib

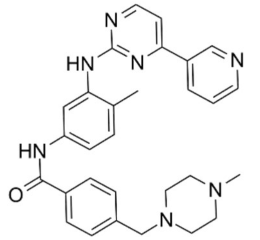

Tucidinostat

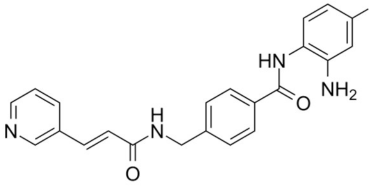

Supplementary Fig. S5

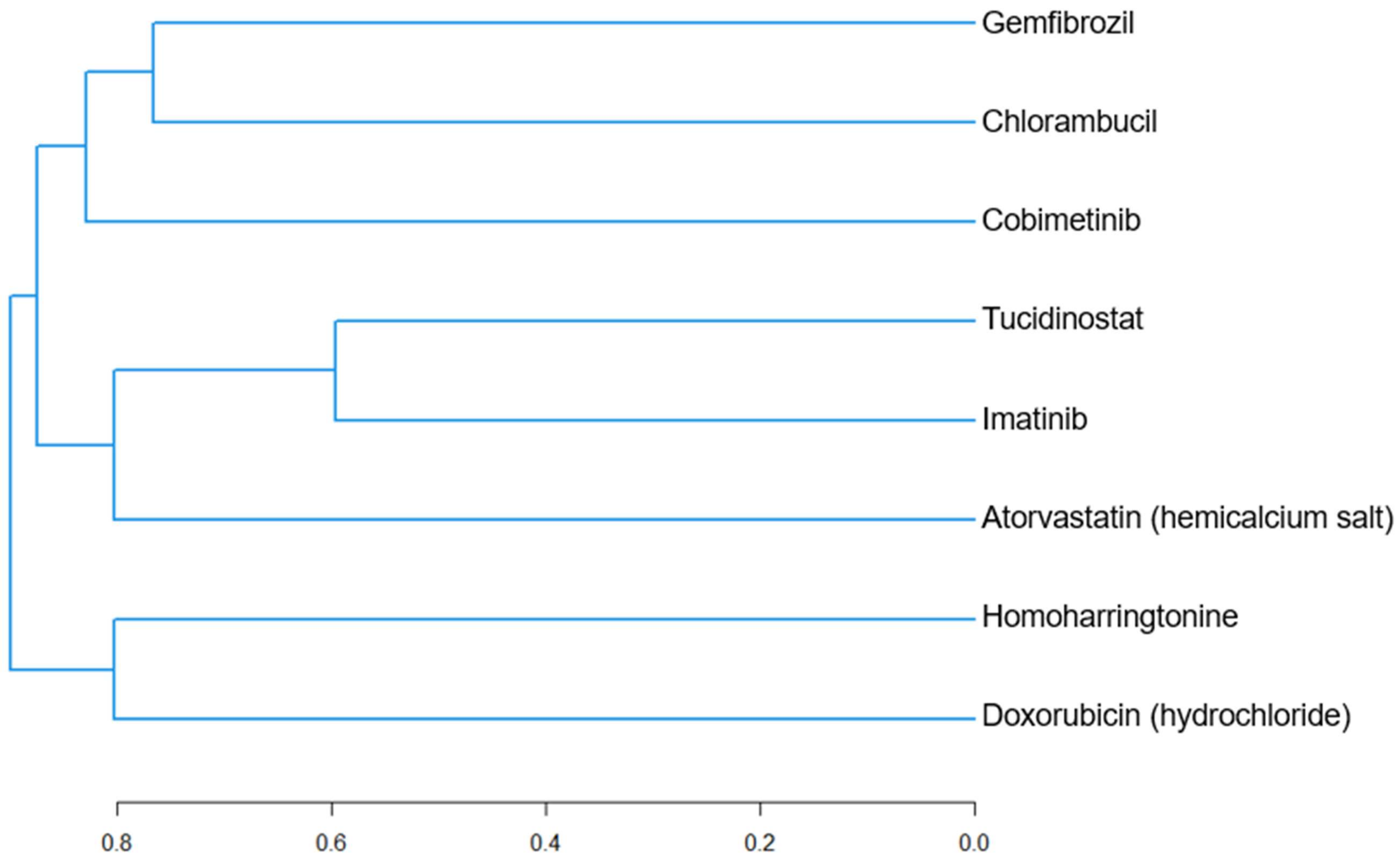

Supplementary Fig. S6

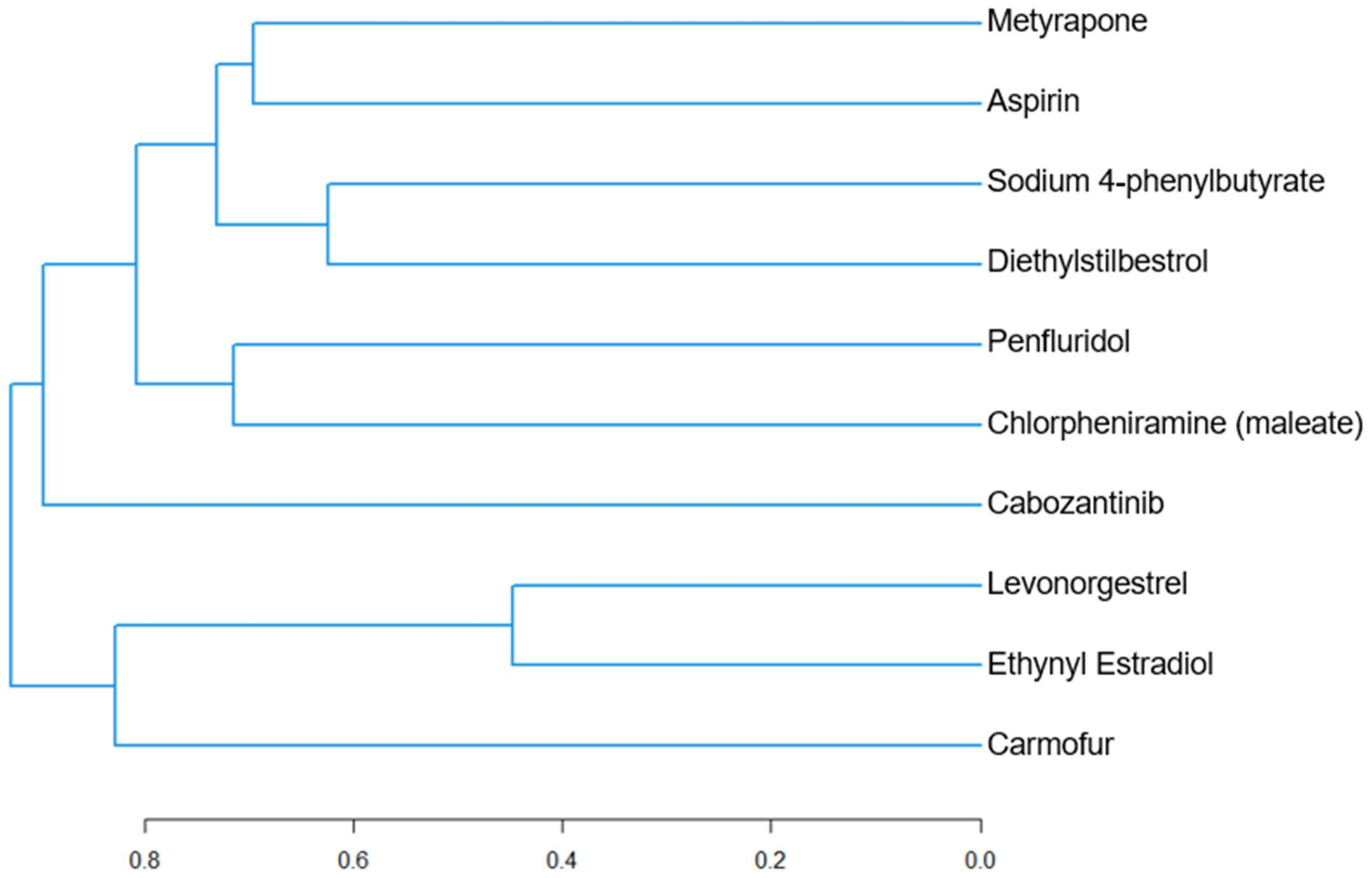

Supplement: Supplementary file 1 [file cells-13-00517-s001.zip › cells-2868073-supplementary.pdf]
